# Examining the influence of socio-economic factors on ultra-processed food consumption patterns of UK adolescents

**DOI:** 10.1017/S136898002510075X

**Published:** 2025-07-25

**Authors:** Rebecca Brody, Zoé Colombet, Esther van Sluijs, Yanaina Chavez-Ugalde

**Affiliations:** 1 MRC Epidemiology Unit, University of Cambridge, School of Clinical Medicine, Cambridge, United Kingdom; 2 Department of Public Health and Policy, University of Liverpool, Liverpool, United Kingdom; 3 NIHR School for Public Health Research, Newcastle, UK

**Keywords:** Ultra-processed food, Adolescence, National Diet and Nutrition Survey, Socio-economic determinants, Socio-demographic determinants

## Abstract

**Objective:**

Ultra-processed food (UPF) consumption varies with socio-economic status (SES) in adults, and evidence suggests that similar patterns exist in adolescents. However, the relationship remains understudied in this critical developmental group. This study aimed to further characterise adolescent UPF consumption and its relationship with SES by exploring dietary patterns within UPF consumption.

**Design:**

Using food-diary data, adolescents’ UPF intake was quantified and categorised. Principal component and clustering analysis were used to identify dietary patterns. Associations of these dietary patterns with socio-demographic characteristics were then analysed.

**Setting:**

Pooled data from the rolling, cross-sectional National Diet and Nutrition Survey, waves 1-to-11 (2008–2019).

**Subjects:**

UK adolescents (11- to18-year-olds) (*n* 3199).

**Results:**

Three UPF dietary patterns were identified: (i) the ‘Restrictive’ pattern, which included the lowest total consumption of UPF (95 % CI: 33·1, 34·9 % g/d), but elevated consumption of UPF often perceived as healthy, was associated with adolescents of a higher SES; (ii) the ‘Permissive’ pattern included 61·6 % g/d (95 % CI: 60·3, 63·0 % g/d) total UPF, dominated by ‘ready-to-eat,’ low nutrient-density UPF, and was associated with adolescents of a lower SES and (iii) the ‘Traditional’ pattern had moderate consumption of total UPF (95 % CI: 47·6, 50·9 % g/d) with higher intake of UPF used in home-cooking and had less distinct associations with SES.

**Conclusion:**

Results suggest that SES impacts both the amount and type of UPF consumed by adolescents in the UK, underscoring the importance of this factor when designing interventions. Distinct dietary patterns within adolescents’ high UPF diets have potential behavioural, nutritional and health implications.

## Introduction

As technology and globalisation have progressed, diets worldwide have become increasingly processed and ultra-processed^(1)^. Ultra-processed foods (UPF), as defined by the Nova food classification system, are industrially manufactured food products that include deconstructed and modified food components, combined with a variety of chemical additives^(2)^. Examples include sugar-sweetened soft drinks, chips and crisps, hot dogs, confectionery and pre-prepared meals. UPF are designed to be standardised, attractive and hyper-palatable. They are also often mass produced by transnational corporations, which commit significant resources to packaging, marketing and distributing these foods, making UPF ubiquitous, low-cost, convenient and desirable^(2,3)^. UPF now contribute up to 50 % of the total energy intake in high-income countries (i.e. Australia, Canada and United States), with the United Kingdom (UK) population reported to have one of the highest levels globally of UPF consumption^(4)^. Evidence has also begun to link higher UPF consumption with adverse health outcomes. Review-level evidence of prospective and cross-sectional studies show associations of increased UPF intake with poor nutrition, overweight, obesity, type II diabetes, CVD, cancer, irritable bowel syndrome, depression and mortality^(3,5)^.

Adolescents represent a special area of concern when considering the potential impacts of UPF. This age group has the highest consumption of UPF in the UK, with 68 % of the total energy intake of adolescents coming from UPF^(6)^. Similar patterns have been found in the US, Brazil, Colombia, Mexico, France and Australia, with adolescents reportedly having 5–30 % higher absolute UPF consumption compared with the adult population^(4,5)^. Adolescents are believed to be especially vulnerable to higher levels of UPF consumption because of their developmental stage, economic dependence on others to provide food, social norms, meal settings and exposure to advertising by food manufacturers^(7)^. As the highest consumers of UPF, adolescents may risk their potential health impacts, such as overweight and obesity^(5)^. This is especially troubling within the context of rising non-communicable diseases levels amongst young people worldwide^(8)^. The harms of UPF may also be magnified within this age group because dietary behaviours formed in childhood influence health and habits over the life course^(7,9)^.

There is further evidence to suggest that UPF consumption varies with socio-economic status (SES). In the UK, adults of a lower SES, as indicated by lower occupational social class, lower household income, lower educational attainment and higher neighbourhood deprivation have increased consumption of UPF^(10,11)^. These disparities also manifest at a regional level. The levels of UPF consumption are lowest in the South of England and London, which have the highest disposable income per capita, while levels are higher in Northern Ireland, Wales and North East England, which have the lowest^(11,12)^. Socio-economic trends in UPF consumption are echoed in other high-income countries^(13–15)^. Within the adolescent sub-group, it appears that there may be similar social patterning of UPF consumption that exists in adult populations. A recent study exploring UPF consumption among youth in the UK found that there was higher UPF consumption among adolescents from lower SES, identified as those with parents in routine and manual occupations^(16)^. However, UPF consumption and its connection to SES remain understudied in adolescents compared with adult populations.

The potential harms of UPF to adolescents are concerning. At the same time, this age may be the most effective time to address UPF consumption. Childhood interventions could allow young people to form new behaviours before entering adulthood, influencing their health and behaviours in the rest of their life and even into future generations^(9)^. Before effective interventions can be structured, there must be a better understanding of adolescent UPF consumption and the factors that influence it. Notably, many current studies focus on the overall quantity or broad categories of UPF consumed by adolescents, rather than the patterns of this consumption^(4,6,7)^. This presents a special challenge as UPF encompass a wide range of food types, which may be eaten in different contexts, for different reasons, and with different impacts on health. This study therefore aimed to characterise UK adolescents’ UPF dietary patterns and their relationship with SES to pave the way for targeted interventions by investigating adolescent UPF dietary patterns for the first time.

## Methods

The current analysis used pooled data from waves 1–11 (2008–2019) of the National Diet and Nutrition Survey (NDNS)^(17)^. This study is reported according to the Strengthening the Reporting of Observational studies in Epidemiology – Nutritional Epidemiology (STROBE-nut).

### Study design and population

NDNS is a rolling, cross-sectional study in the UK that has been conducted annually since 2008. The study is intended to provide information about the food and nutrient intake and nutritional status of the UK population. NDNS further aims to capture how nutrition trends connect to individuals’ socio-demographic characteristics and health outcomes.

NDNS aimed to have a total of 1000 participants, with an equal balance of adults (age > 19) and young people (age ≤ 19), for each study year. Recruitment was carried out using the postcode address file, a list of all known postcode addresses in the UK. Addresses from the postcode address file were grouped into primary sampling units, each representing geographic areas across the UK. Then, a random sample of addresses was drawn from each primary sampling unit. One child and one adult were chosen at random from households at the selected addresses. In order to balance the number of children and adults in the sample, a random subset of the households had only children surveyed. Trained interviewers collected socio-demographic information via interviews and distributed 4-day dietary diaries. Diary data were collected over the course of four consecutive days, with the first day chosen at random and designed to include at least one weekend day (e.g. starting on a Thursday, Friday or Saturday and included both weekend days or starting on a Wednesday to include at least on weekend day).

Further detail on sampling methodology can be found elsewhere^(18,19)^.

NDNS defined seven age groups within the sample: 1·5–3 years; 4–10 years; 11–18 years; 19–64 years; 65 years and over; 65–74 years and 75 years and over. This analysis selected individuals aged 11–18 years to represent the adolescent period^(19)^.

Parental consent was obtained for participants aged 11–15 years, and written informed consent was obtained from participants aged 16–18 years^(18)^. Additional ethical approval for this secondary analysis of anonymised data was not required.

### Dietary assessment

Participants were asked to complete food diaries to record all foods and beverages consumed over the course of four consecutive days, as well as the location and time of consumption. The parents of adolescents ages 11 and 12 were instructed to fill out the food diaries for their children. Four-day food diaries have been validated as an appropriate tool to capture food consumption in this age group^(20)^. Portion sizes were estimated using standard household measures (i.e. tablespoons) or based on nutrition labels. Individuals ages 16 or older were provided with reference photos of portion sizes for commonly consumed foods. For participants younger than 16 (11–15 years of age), a validated young person’s food atlas was used to review portion sizes^(20,21)^. Interviewers checked food diaries during and following the 4-day recording period to encourage quality and completeness. The collection periods were also conducted across different days of the week and seasons to account for seasonal and weekly variations^(18)^.

The food diaries were processed by trained coders using the Diet In Nutrients Out assessment system, incorporating food composition data from the Department of Health’s NDNS Nutrient Database. Whenever possible, meals were broken down into constituent foods and beverages, and each was coded as a separate entry. The coding process is described in further detail elsewhere^(20)^.

### Food classification

All dietary data from years 1–11 of the study were combined, resulting in a total of 1 531 636 recorded consumed food items, including 4944 unique types of food. Each food was further classified based on level of processing using the Nova (not an acronym) food classification system, developed by Monteiro and colleagues^(2)^. The Nova scale assigns foods to one of four categories: unprocessed or minimally processed foods (Group 1), processed culinary ingredients (Group 2), processed foods (Group 3) and UPF (Group 4)^(2)^. Further details on the Nova classification system can be found in the Supplemental Material. Each of the unique foods recorded in years 1–11 had been previously categorised into Nova categories by two independent researchers (YCU, ZC), with a 96·9 % level of agreement^(22)^.

The food items classified as Nova Group 4 were further sub-categorised by RB, with secondary verification by ZC. An initial list of UPF sub-types was developed with reference to pre-existing literature and food sub-categories present in the NDNS dataset^(6,7,23,24)^. As classification was conducted, the list was refined, and foods were re-categorised as needed. New sub-types were created when there were over five foods that did not fit within an existing category. Sub-types were removed or collapsed when this was not the case. This resulted in a final set of 34 UPF sub-types. A set of rules and example foods were created to guide classification (see online supplementary material, Supplemental Table 1).

The primary outcome of interest was daily UPF intake, in terms of daily relative energy (percentage energy from UPF per day) and daily relative weight (percentage weight from UPF per day)^(5)^. This was calculated for overall UPF consumption and each of the thirty-four UPF sub-types.

### Socio-demographic characteristics

Age, sex, ethnicity, parental occupation status, housing tenure and region were included as socio-demographic characteristics based on the variables present in years 1–11 of the NDNS dataset. Each categorical variable was given a designated reference level based on order of appearance in the data dictionary. For parental occupation, the eight-level version of the National Statistics Socio-economic Classification scale was converted into the three-level version, as described, to aid interpretability^(18,25)^. ‘Never worked’ was retained as a separate group, resulting in a total of four categories (see results section Table 1)^(25)^. Household tenure was also collapsed from six categories into four to aid interpretability, following the 2021 UK Census standards^(26)^.

Certain socio-demographic characteristics that were not applicable to adolescents, such as occupation, were recorded for the household reference person, rather than the adolescent themselves. The household reference person was selected as the individual whose name the household’s accommodation was owned or rented under. In the case where this criteria applied to two or more adults in the household, the individual with the highest income was chosen^(27)^.

### Statistical analysis

For the current analysis, only participants that had completed at least three of the four food diary days were included, as per the NDNS data collection methodology. No participants within the selected age range were excluded on this basis, as all had completed 4 days. Complete-case analysis was also used, so any participants that were missing date for at least one of the variables of interest were excluded. Descriptive statistics for the participants who had missing data were completed separately (see online supplementary material, Supplemental Table 2).

All data analysis was performed in RStudio version 2022.07.1 with an RMarkdown format^(28)^.

Study weights provided by NDNS were used in all analyses for the adolescent sub-sample to account for sampling and non-response bias^(18)^.

### Descriptive analysis

The average daily intake of total UPF and each UPF sub-type, in terms of relative energy (% kcal/day) and relative weight (% g/day) from UPF, was calculated for the overall sample, as well as each of the socio-demographic subgroups.

### Identifying ultra-processed foods dietary patterns

To identify patterns of UPF intake, principal component analysis (PCA) and clustering analysis were employed. First, a weighted PCA was applied to simplify the highly dimensional dataset. Two datasets describing the sample’s daily relative energy and mass from the UPF sub-types were normalised, then PCA was applied. Following the first round of PCA, UPF sub-types having factor loading coefficient magnitude under 0·20 for these principal components (PC) were then removed to improve the explanatory power of the PC^(29)^. PCA was then repeated with data for the remaining twenty-two UPF sub-types for daily relative energy and twenty-three sub-types for daily relative weight (out of the initial thirty-four categories) (see online supplementary material, Supplemental Tables 6, 7). The Kaiser criterion (eigenvalues ≥ 1) and Scree plots were used to select meaningful PC, yielding three PC, which were selected for the daily relative energy and four for daily relative weight (see online supplementary material, Supplemental Figure 1)^(30)^.

The suitability of the data for clustering was confirmed using the Hopkins test^(31)^. Hierarchical clustering analysis was then performed on the three PC generated from the data in order to identify potential dietary patterns within the data. The optimal number of clusters was selected automatically at the point where inertia was maximised^(32)^. Graphical observation of the dendrogram was used to verify the appropriate number of clusters. Cluster analysis yielded three groups, interpreted as dietary patterns, which were described and labelled according to their pattern of UPF sub-types.

### Dietary pattern analysis

Once clusters were generated, the average UPF consumption of the individuals in the cluster was described in terms of average daily relative energy (% kcal/day) and relative weight (% g/day) from all UPF and each UPF sub-type. These clusters of UPF intake were interpreted as dietary patterns and labelled according to their main UPF sub-type intakes^(33)^. The socio-demographic characteristics of individuals in each cluster were described by calculating the percentage of sample (%*n*) for each socio-demographic sub-group, as well as the average age in the clusters.

Logistic regression was used to explore associations between socio-demographic characteristics and the UPF dietary patterns. Membership in each cluster was re-coded as a binary variable (1 if an individual was in the cluster, 0 if not). OR therefore describe the odds of someone belonging to a specific UPF dietary pattern *v*. not. Univariate analysis was conducted with each of the variables of interest (age, sex, occupation, housing tenure, ethnicity and region) to confirm their significance. Then, in a single, multivariate logistic model, cluster membership was regressed against all of the variables simultaneously to account for potential shared confounding factors. The model was further adjusted for individuals’ total dietary intake (in terms of total daily kilocalories or total daily grams) and overall level of UPF intake (in terms of % kcal from non-UPF/day or % g from non-UPF/day) because these features were independently associated with some socio-demographic characteristics. A *P* value < 0·05 was considered significant.

## Results

### Participant characteristics

Analyses included participants from waves 1–11 of NDNS between ages 11–18 at the time of the survey, resulting in an initial sample of 3270 individuals. Of this sample, seventy-one individuals (2·2 %) had missing data for at least one variable of interest, and these individuals were excluded from analysis, resulting in a final complete-case sample size of 3199. Online supplementary material, Supplemental Table 2 presents the descriptive statistics of those excluded for missing data.

The sample of complete cases had a weighted average age of 14·5 years (95 % CI: 14·45, 14·55 years), with an approximately even number of men and women. The majority of participants were White (82·1 %). Within the sample, 41·3 % of adolescents had parents employed in higher managerial, administrative or professional occupations; 22·2 % in an intermediate occupation; 33·1 % in a routine and manual occupation and 3·4 % that had never worked. Around half lived in a house owned with mortgage (53·7 %) or rented through social housing (21·5 %). The most represented region was Southern England (43·3 %), followed by Northern England (23·6 %) and Central and Midlands England (17·3 %). The full socio-demographic characteristics are described in Table 1.

**Table 1.** Socio-demographic characteristics of adolescents (ages 11–18) from years 1–11 of the NDNS study with complete data for all variables of interest (*n* 3199)

Socio-demographic characteristic		Weighted %*n* *	95 % CI
Age			
	11	12·3	10·9, 13·9
	12	12·7	11·3, 14·3
	13	12·5	11·1, 14·1
	14	13·0	11·6, 14·6
	15	11·1	9·8, 12·5
	16	14·7	13·1, 16·5
	17	13·9	12·4, 15·6
	18	9·8	8·5, 11·1
Sex			
	Female	48·7	46·5, 51·0
	Male	51·3	49·0, 53·5
Ethnicity			
	White	82·1	80·1, 83·9
	Asian or Asian British	9·2	7·8, 10·7
	Black or Black British	3·9	3·1, 5·0
	Mixed ethnic group	2·9	2·2, 3·8
	Any other group	2·0	1·4, 2·8
Parental occupation			
	Higher managerial, administrative and professional occupations	41·3	39·1, 43·6
	Intermediate occupations	22·2	20·4, 24·2
	Routine and manual occupations	33·1	31·0, 35·2
	Never worked	3·4	2·6, 4·4
Housing tenure			
	Own outright	12·6	11·3, 14·2
	Own with mortgage	53·7	51·4, 55·9
	Rent privately	12·2	10·8, 13·8
	Rent social housing	21·5	19·6, 23·5
Region			
	England: North	23·6	21·7, 25·6
	England: Central/Midlands	17·3	15·7, 19·1
	England: South (incl. London)	43·3	41·0, 45·6
	Northern Ireland	3·2	2·9, 3·6
	Scotland	7·8	6·6, 9·1
	Wales	4·8	4·2, 5·5

NDNS, National Diet and Nutrition Survey.

* Values are reported as weighted percentage of sample (%*n*) with 95 % CI. Percentages and means are weighed based on non-selection and non-response survey weights provided by NDNS year 2008–2019.

### Ultra-processed foods sub-type intake

**Figure 1.**
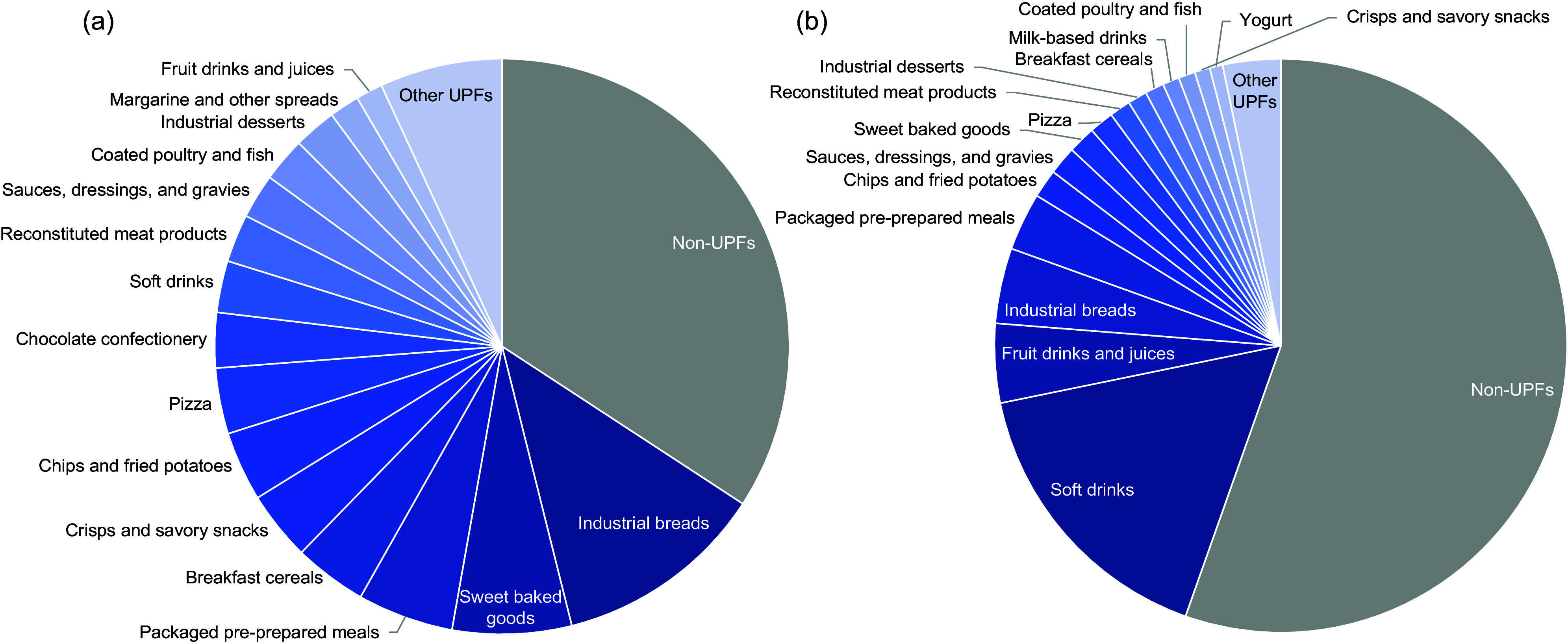
Average daily relative energy (% kcal/day) (a) and weight (% g/day) (b) from non-UPF (gray) and all UPF sub-types (shades of blue) in adolescents (11–18 years old) from years 1–11 of the NDNS study (*n* 3199). NDNS, National Diet and Nutrition Survey; UPF, ultra-processed food.

On average, the adolescents’ daily UPF intake was 65·8 % kcal/d and 44·6 % g/d (Figure 1) (see online Supplemental Table 3 for more detail). In terms of daily relative energy, industrial bread was the most highly consumed UPF sub-type by adolescents, contributing an average of 12 % kcal/d. This was followed by sweet baked goods (6·8 % kcal/d), packaged pre-prepared meals (5·4 % kcal/d) and breakfast cereals (4·1 % kcal/d) (Figure 1(a)) (see online supplementary material, Supplemental Table 4).

In terms of daily relative weight, soft drinks were, by far, the most consumed UPF sub-type, contributing an average of 16 % g/d. This was followed by fruit drinks and juices (4·5 % g/d), industrial breads (4·3 % g/d) and packaged pre-prepared meals (3·3 % g/d) (Figure 1(b)) (see online supplementary material, Supplemental Table 5).

### Ultra-processed foods dietary patterns

The PCA of the data describing daily relative energy from UPF sub-types yielded three informative PC, which collectively explained 20·4 % of the variation in the data (see online supplementary material, Supplemental Figure 1(a)). The PCA of the data describing daily relative weight from UPF sub-types yielded four informative PCs, collectively explaining 25·7 % of the variation in the data (Supplemental Figure 1(b)) (see online supplementary material, Supplemental Tables 7,8). Thus, the results of the clustering analysis performed on the PCA results for daily relative weight are discussed in further depth because these patterns were able to explain more of the variation in the data. However, clustering analysis of the PCA from daily relative energy data was conducted in tandem and yielded similar results (See Supplemental material Tables 6 and 9).

Cluster analysis of the PCA results revealed three groups within the data. The UPF sub-type intake for these clusters was described and interpreted as three dietary patterns, which were labelled as ‘Restrictive,’ ‘Traditional’ and ‘Permissive.’ These patterns represented 50·6 %, 17·9 % and 34·4 % of the sample, respectively.

**Figure 2.**
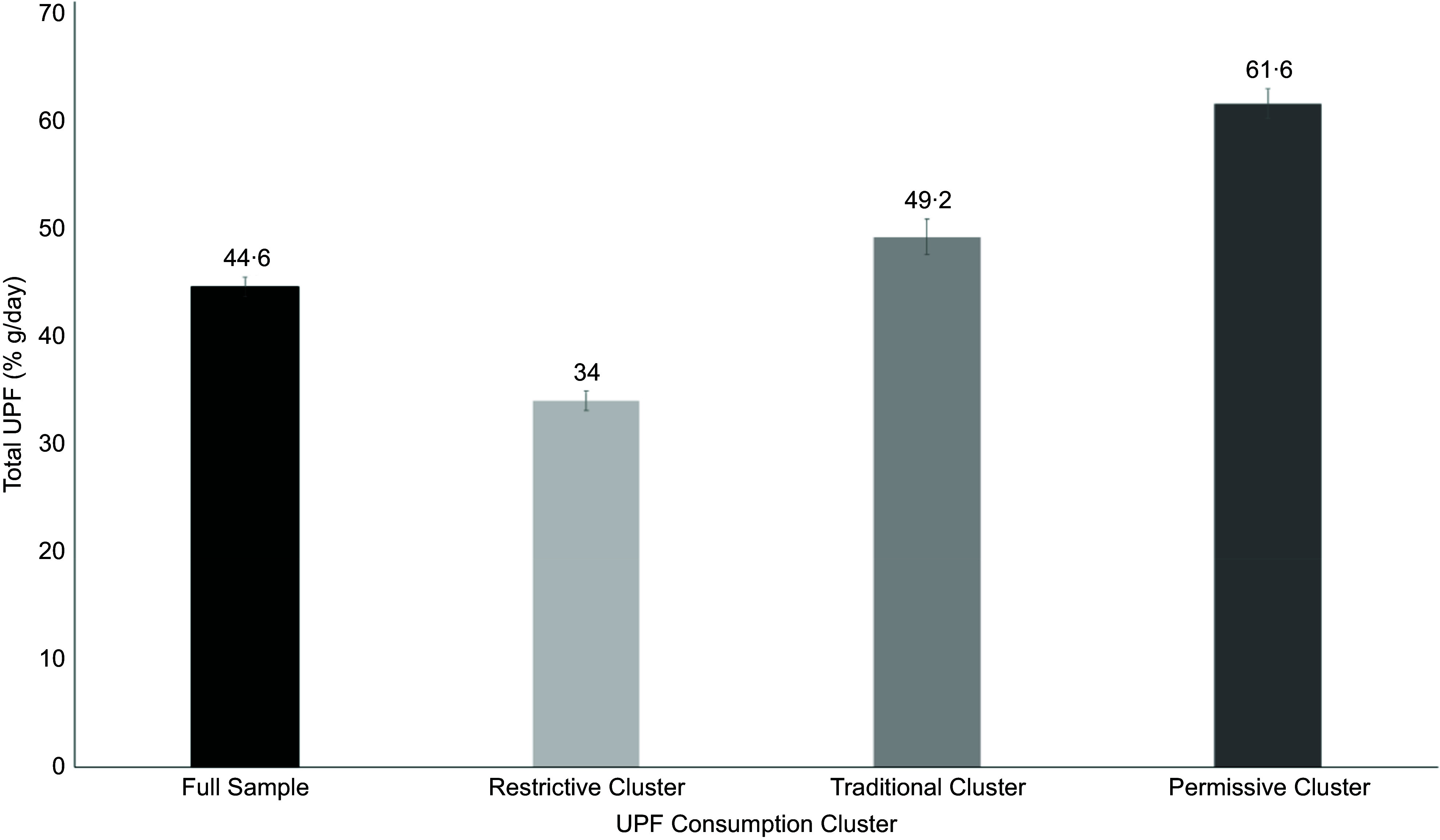
Average daily total relative weight from UPF (% g/day) in adolescents (11–18 years old) from years 1–11 of the NDNS study for the full sample (*n* 3199) and each identified dietary cluster (using PCA and cluster analysis) – Restrictive, Traditional and Permissive. The displayed categories from left to right are Full Sample, Restrictive Cluster, Traditional Cluster and Permissive Cluster. Error bars represent 95 % CI. NDNS, National Diet and Nutrition Survey; PCA, principal component analysis; UPF, ultra-processed food.

There were differences in the average total amount of UPF consumed by adolescents in each of the clusters. Adolescents in the Restrictive cluster consumed the lowest total UPF, with an average of 34·0 % g/d. Adolescents in the Traditional cluster consumed an average of 49·2 % g/d. The highest level of total UPF was consumed by adolescents in the Permissive cluster, with 61·6 % g/d coming from UPF (Figure 2).

**Figure 3.**
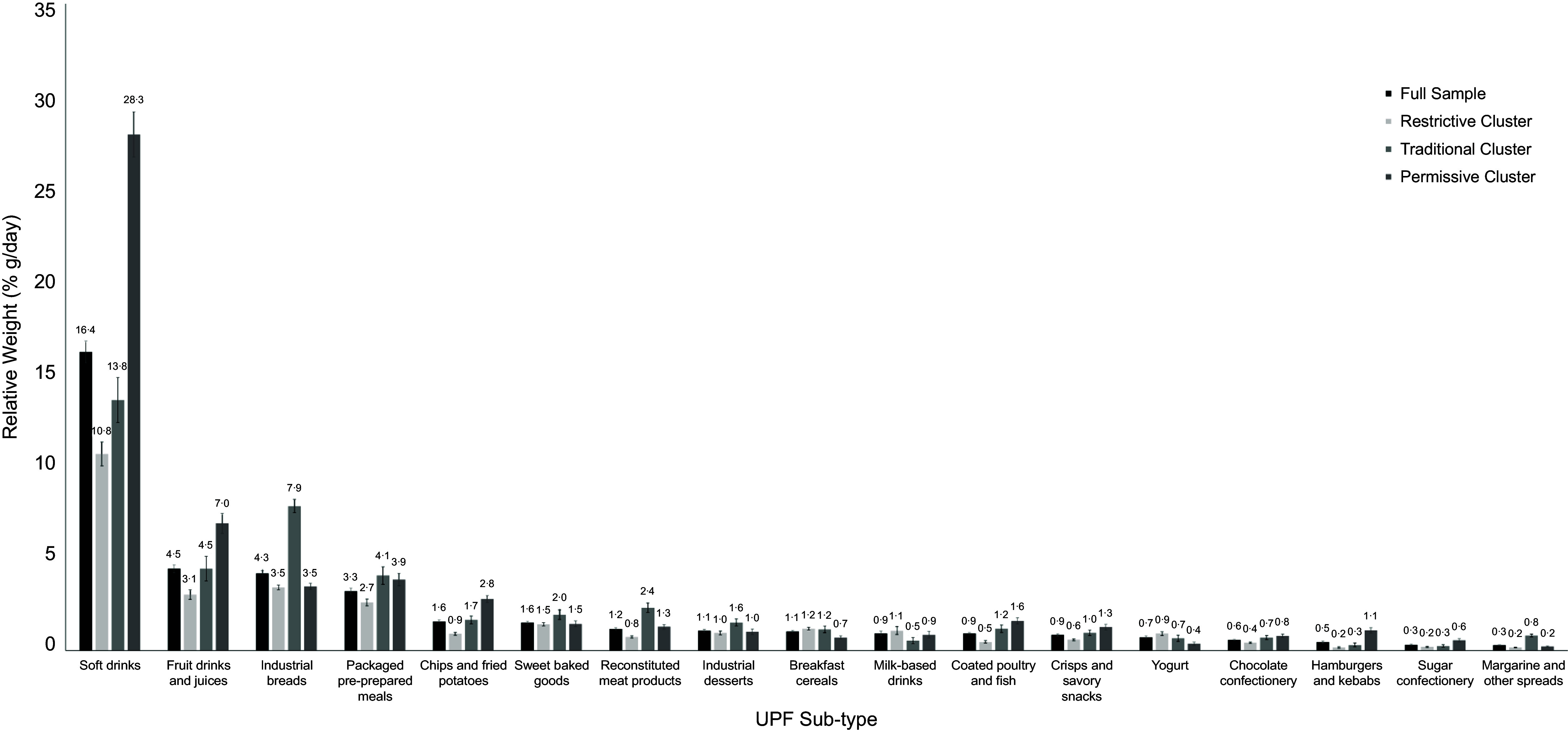
Average daily relative weight from UPF sub-types (% g/day) in adolescents (11–18 years old) from years 1–11 of the NDNS study for the full sample (*n* 3199) and each identified dietary cluster (using PCA and cluster analysis) – Restrictive, Traditional, and Permissive. The displayed categories from the left to right are Full Sample, Restrictive Cluster, Traditional Cluster and Permissive Cluster (see key). The displayed UPF sub-types differed significantly from the full sample average for at least one cluster. Error bars represent 95 % CI. NDNS, National Diet and Nutrition Survey; PCA, principal component analysis; UPF, ultra-processed food.

Each dietary pattern was also distinguished by UPF sub-types consumed at a significantly higher or lower levels compared with the sample average (*P* < 0·05) (Figure 3) (see online supplementary material, Supplemental Table 8). Adolescents in the Restrictive cluster had a higher consumption of breakfast cereals, meat alternatives and yogurt and lower consumption of most other UPF sub-types, including hamburgers and kebabs, coated poultry and fish, chips and fried potatoes and margarine and other spreads. Adolescents in the Traditional cluster had high intakes of sweet baked goods, industrial desserts, industrial bread, margarine and other spreads, reconstituted meat products and packaged pre-prepared meals, while having a lower-than-average intake of dairy alternatives, meat alternatives, meal replacements and sports foods and milk-based drinks. Lastly, consumption patterns in adolescents in the Permissive cluster were characterised by higher intake of chips and fried potatoes, coated poultry and fish, hamburgers and kebabs, packaged pre-prepared meals, crisps and savoury snacks, soft drinks, fruit drinks and juices, chocolate confectionery and sugar confectionery, but lower-than-average consumption of breakfast cereals, crackers and savoury biscuits, dairy alternatives, industrial breads, margarine and other spreads, meat alternatives, mixes and yogurt.

When using relative energy data (see online supplementary material, Supplemental Table 9), the only differences seen were regarding the intake of some desserts and alcohol within the dietary patterns. The Permissive cluster was characterised by a higher level of consumption of these UPF sub-types, compared with the Restrictive and Traditional clusters.

### Association of socio-demographic characteristics with ultra-processed foods dietary patterns

Table 2 presents associations between socio-demographic characteristics and clusters based on the daily relative weight data. Analyses with clusters from the daily relative energy intake were largely similar (see online supplementary material, Supplemental Table 10).

**Table 2.** Associations of socio-demographic characteristics of adolescents (11–18 years old) from years 1–11 of the NDNS study with each identified dietary pattern (based on PCA and cluster analysis) based on the average daily relative weight from each of the UPF sub-types (% g/day)

Socio-demographic characteristic	Restrictive (*n* 1618)			Traditional (*n* 572)			Permissive (*n* 1009)		
	OR*	95 % CI	*P* value†	OR*	95 % CI	*P* value†	OR*	95 % CI	*P* value†
Age (years)	0·99	0·99, 1·00	0·20	0·99	0·99, 1·00	0·05	1·01	1·01, 1·02	0·001
Sex									
Female (ref)									
Male	0·96	0·92, 0·99	0·02	1·08	1·05, 1·12	< 0·001	0·96	0·93, 1·00	0·038
Parental occupation									
Higher occupations (ref)									
Intermediate occupations	0·97	0·93, 1·02	0·3	1·03	0·98, 1·07	0·22	1·00	0·96, 1·05	0·94
Routine and manual occupations	0·93	0·89, 0·97	< 0·01	1·04	1·00, 1·09	0·05	1·03	0·99, 1·08	0·13
Never worked	0·88	0·77, 0·99	0·04	1·02	0·91, 1·14	0·75	1·12	1·00, 1·25	0·04
Housing tenure									
Own outright (ref)									
Own with mortgage	0·96	0·91, 1·02	0·17	1·01	0·97, 1·06	0·60	1·03	0·98, 1·07	0·25
Rent privately	0·97	0·91, 1·04	0·44	0·98	0·93, 1·04	0·49	1·05	0·99, 1·12	0·13
Rent social housing	0·90	0·84, 0·96	< 0·01	1·04	0·98, 1·10	0·24	1·07	1·01, 1·14	0·03
Ethnicity									
White (ref)									
Asian or Asian British	1·09	1·01, 1·18	0·01	0·94	0·89, 1·01	0·09	0·97	0·91, 1·02	0·25
Black or Black British	1·06	0·95, 1·20	0·29	0·95	0·85, 1·05	0·31	0·99	0·90, 1·09	0·88
Mixed ethnic group	1·04	0·94, 1·16	0·43	0·91	0·84, 0·99	0·03	1·05	0·95, 1·16	0·36
Any other group	1·11	0·98, 1·25	0·10	0·86	0·81, 0·92	< 0·001	1·05	0·94, 1·17	0·41
Region									
England: North (ref)									
England: Central/Midlands	1·01	0·96, 1·08	0·63	1·00	0·95, 1·06	0·95	0·98	0·93, 1·04	0·56
England: South (incl. London)	1·02	0·97, 1·07	0·40	0·99	0·95, 1·03	0·60	0·99	0·95, 1·04	0·69
Northern Ireland	0·95	0·90, 1·01	0·09	1·01	0·96, 1·07	0·75	1·04	0·98, 1·09	0·19
Scotland	0·93	0·86, 1·01	0·07	0·99	0·92, 1·06	0·66	1·09	1·00, 1·18	0·04
Wales	0·94	0·88, 1·00	0·05	1·02	0·96, 1·08	0·62	1·05	0·98, 1·12	0·19

NDNS, National Diet and Nutrition Survey; PCA, principal component analysis; UPF, ultra-processed food.

* OR and 95 % CI are reported.

† Multivariate logistic regression assessed likelihood of membership of cluster (*v*. not) for each socio-demographic characteristic separately. Models were mutually adjusted for all socio-demographic characteristics, as well as for total weight intake and overall relative weight from UPF. Age was considered as a continuous variable. Each of the other categorical variables was compared with a designated reference level (sex –– Female; ethnicity – White; parental occupation – higher managerial, administrative and professional occupations (higher occupations); housing tenure – own outright; region –England: North).

Membership in the Restrictive cluster was slightly more common for female adolescents, with this group being 4·0 % (*P* = 0·02) more likely to belong to the cluster than males. Adolescents with parents in routine and manual occupations and those with parents who had never worked were 7·0 % (*P* < 0·01) and 12 % (*P* = 0·04) less likely to belong in the Restrictive cluster than those whose parents were in higher managerial, administrative and professional occupations, respectively. Adolescents living in social housing were 10 % (*P* < 0·01) less likely to belong to the cluster compared to adolescents living in homes owned by their families. Lastly, Asian or Asian British adolescents were 9 % (*P* = 0·01) more likely to belong to this cluster than White adolescents (Table 2).

For the Traditional cluster, there were fewer distinct associations with socio-demographic features. Male adolescents were 8 % (*P* < 0·001) more likely to follow this dietary pattern than female adolescents. Adolescents of mixed or other ethnic identity were 9·0 % (*P* = 0·03) and 14 % (*P* < 0·001) less likely to belong to the Traditional cluster than White adolescents, respectively (Table 2).

Finally, adolescents with parents who had never worked were 12 % (*P* = 0·04) more likely to belong to the Permissive cluster. Adolescents living in social housing were also 7 % (*P* = 0·03) more likely to belong to this cluster. Adolescents from Scotland were 9·0 % (*P* = 0·04) more likely to belong to this group than adolescents from Northern England, which was the only association that existed with region (Table 2).

## Discussion

The current analysis characterised adolescent UPF intake, describing UPF dietary patterns and exploring their associations with socio-demographic characteristics in a representative sample of UK adolescents. The work identified three novel UPF dietary patterns, which were labelled: ‘Restrictive,’ ‘Traditional’ and ‘Permissive.’ Overall, the findings of this analysis demonstrate the importance of evaluating dietary patterns within UPF consumption to understand social determinants underlying these dietary behaviours. They further indicate that adolescents of lower SES have both higher overall levels of UPF consumption and are more likely to consume ready-to-eat, high in fat, sugar and salt (HFSS) and UPF across meal contexts. As a result, adolescents of a low SES may face both more risk from UPF, as well as a combined threat from the other nutritional features of HFSS foods they are consuming. This trend points to the concerning possibility that UPF may perpetuate and worsen non-communicable disease disparities amongst adolescents. This work is needed to inform public health policies, especially as the UK adolescent UPF consumption reaches over 60 % of the daily total energy intake.

The Permissive diet was associated with individuals of a lower SES, as indicated by higher likelihood of adolescents with parents who had never worked or living in social housing belonging to this cluster. In contrast, the Restrictive pattern was most associated with individuals of a higher SES, as shown by adolescents with parents in higher managerial, administrative and professional occupations and those living in homes owned by their families being more likely to belong to this cluster. The Traditional dietary pattern had less distinct connections to SES. However, this pattern was associated with younger, male adolescents and was less common amongst adolescents from other, non-White ethnic groups.

The three dietary patterns identified had clear differences in the total amount of UPF consumed. Adolescents in the Permissive cluster consumed the highest amount of total UPF within their diet, consuming an average of 17 % g/d more UPF than the sample average. The Traditional cluster had a total UPF consumption similar to that of the sample average. The Restrictive cluster had a total UPF consumption 10·6 % g/d less than the sample average. This is consistent with other studies, which have found that SES is associated with the total amount of UPF consumed by adolescents^(16)^.

The dietary clusters also had distinct combinations of UPF sub-types, with potential unique contexts of consumption and nutritional value. The Restrictive pattern was characterised by consumption of UPF often eaten for breakfast (e.g. breakfast cereals) or as part of specialised diets, such as vegetarianism (e.g. meat replacements). Vegetarianism has been found to be associated with increased consumption of UPF, especially when the diet is begun at a younger age^(34)^. However, this behaviour has not been studied specifically in an adolescent population. Ultra-processed meat replacements can carry the same risks as all UPF, but vegetarian’s overall diet quality may be better when quantified through other metrics, such as the healthy and unhealthy plant-based diet indices^(34)^. These food items are also more likely to be perceived as healthy by consumers^(35–40)^.

In contrast, the Permissive pattern included UPF that are eaten for lunch, dinner, snacks, desserts and ‘on-the-go’ (e.g. hamburgers and kebabs). These foods are often purchased ready-to-eat from grocery stores, convenience stores or take-away restaurants. Eating location has previously found to be associated with both the total quantity and types of UPF consumed by adults and adolescents^(41)^. Many of the characteristic components of the Permissive diet, such as crisps and sweetened soft drinks, are products commonly HFSS with a low nutrient-density and have been linked to non-communicable diseases independently of their level of processing^(38–40)^. Further, these foods may be more likely to be viewed as unhealthy by consumers due to existing HFSS messaging, as well as perceived lack of nutritional value and association with increased risk of weight-gain^(35–38)^.

Finally, the Traditional pattern included UPF sub-types that are often combined with other ingredients to make a meal and may be used in more traditional home cooking (e.g. margarine and reconstituted meat products). This may indicate cultural patterning of this diet rather than socio-economic, such as a cultural emphasis on cooking and sharing meals in the home^(42)^. This is further indicated by the stronger associations of this group with gender, ethnicity and age, rather than economic markers.

The behaviours underlying UPF dietary patterns could be a result of economic and social influences. Certain types of UPF being more affordable or more accessible for families experiencing time scarcity or food insecurity^(14,43)^. UPF consumption could also be influenced by a specific lack of information surrounding UPF and differing social norms. Qualitative studies have revealed that community practices, as well as perceptions of the healthiness of different UPF, determined the quantity and context that parents gave their children UPF^(44)^. This, in turn, can influence adolescents’ own decisions regarding UPF^(44)^.

### Strengths and limitations

To the best of our knowledge, this is the first study to characterise data associated with adolescents UPF dietary patterns in a representative sample of UK adolescents. Due to the consistent dietary data collection methods, the data across waves 1–11 in NDNS could be combined providing a relatively large sample size. The use of study weights in all analyses helps to account for non-response and sampling biases allowing for the study results to be generalisable to the UK adolescent population^(18)^.

Food diaries are a flexible dietary assessment method that can be used across a wide age range and provide information about usual consumption habits^(20)^. As with all methods of dietary assessment, there is the potential of misreporting of energy intake. It has been found that for NDNS specifically, misreporting by young people has been increasing over time^(45)^. In addition, adolescents under the age of 12 had dietary diaries filled out by their caretakers. There could also be a biasing in data collection due to the use of consecutive days of recording by over or under-representing consumption on a particular day (e.g. weekdays *v*. weekends).

Further, information on the level of physical activity in the NDNS dataset is only available for adolescents ages 16–18 and thus was not factored into analyses. Future analyses should include adjustments for the misreporting of energy intake and the level of physical activity to verify the consistency of the associations observed in this study. For the classification of the dietary data using the Nova system, there was a high level of agreement amongst researchers (97 %)^(22)^. However, the possibility of some misclassification of foods into Nova categories and sub-categories cannot be excluded^(2,7,23)^.

Two key indicators of SES, equivalised household income and the index of multiple deprivation, were not used. There were inconsistencies in reporting equivalised household income in the NDNS across survey years. The index of multiple deprivation is calculated in different ways in each country of the UK, preventing use in the aggregate dataset. It is also difficult to directly measure the SES of the adolescents, therefore parental measures were used as a proxy. However, the alternate measures of SES employed in the analysis have been found to be strong indicators of adolescent SES^(46)^.

The use of PCA and clustering analysis allowed for the simplification of a highly dimensional dataset and the discovery of dietary patterns. These methods may be limited in their explanatory power, but the PC generated through PCA were able to explain an amount of variation in the data similar to that of other studies exploring dietary patterns^(47,48)^. Lastly, the magnitude of associations between socio-demographic characteristics and the dietary patterns were relatively small, potentially due to smaller sample sizes in some sub-categories. These associations should be confirmed in additional datasets.

### Future research and policy implications

Based on the findings of this analysis, interventions and policy surrounding UPF should incorporate an understanding of adolescent SES to target specific behaviours underlying consumption. Approaches should also limit the potential increased burden of detrimental health effects on adolescents of a lower SES. This could involve interventions in schools or other places where social services are provided to disadvantaged adolescents and their families, such as institutional bans on UPF in schools and hospitals^(49)^.

These solutions must be combined with structural level changes as well, given the complex economic and socio-political context that surrounds UPF. Currently, UPF may be one of the few sources of affordable and accessible food for some families. Strategies could involve promoting affordability of MPF by taxes on UPF and subsidies for MPF^(49)^. The accessibility of MPF can also be increased by promoting the use of less processed food in the home but also in restaurants, take-aways and grocery stores^(50)^. The simultaneous use of these methods will gradually impact the broader cultural and social norms influencing UPF consumption by adolescents, as well as reduce UPF consumption across life stages.

In addition, further analyses should be conducted with the current and additional datasets to validate and expand on the findings of this analysis, such as exploring broader dietary patterns including unprocessed and minimally processed foods. It is also essential to consider the specific associations between the UPF dietary patterns and health outcomes of adolescents.

The ultimate goal is the creation of a clear set of guidelines that can guide individuals to reduce consumption of UPF and increase consumption of minimally processed foods, with consideration of the factors that lead young people to eat UPF.

## Conclusion

The results of the current analysis reaffirm the importance of addressing UPF consumption among adolescents in the UK. Adolescents have the highest consumption of UPF overall and has distinct dietary patterns across socio-demographic groups. This indicates the importance of further exploring and addressing the consumption and dietary patterns of UPF particularly amongst low SES adolescents, who have the highest consumption of UPF, as well as a dietary pattern including more generally unhealthy, ready-to-eat UPF, putting them at higher potential health risk. This study supports the importance of designing targeted interventions and policies to address UPF consumption in adolescents to limit the potential detrimental health impacts on this age group.

## Supporting information

Brody et al. supplementary material Brody et al. supplementary material

## Supplementary material

For supplementary material accompanying this paper visit https://doi.org/10.1017/S136898002510075X
