# Supplementary material for: Examining the influence of socio-economic factors on ultra-processed food consumption patterns of UK adolescents
Source: Public Health Nutr. 2025 Jul 25;28(1):e140. doi: 10.1017/S136898002510075X (PMC12516625; doi:10.1017/S136898002510075X)
Supplement: Brody et al. supplementary material [file S136898002510075Xsup001.docx]

**Supplementary Material**

**Nova Classification System**

Nova Group 1 foods are taken directly from plants, animals, fungi, or other natural sources with no processing or minimal additional processing to allow for safe consumption and storage (e.g. drying, griding, and pasteurizing). Examples include fresh fruit and vegetables, milk, grains, legumes, and meat (Figure 1). Group 2 foods are substances derived from Group 1 foods through additional processing methods, such as pressing and refining, and are often intended for use in cooking rather than direct consumption. These include oil, flour, salt, and butter. Group 3 foods are created by combining Group 1 and 2 foods. They are still considered to contain whole foods but have undergone additional processing to increase their durability or alter taste, texture, or smell. This processing is often simple enough to occur at home, such as smoking fish, canning vegetables in brine, or baking bread (Figure 1)^(2,5)^.

Finally, Group 4 foods, UPFs, result from the combination and further modification of Group 2 foods, with little or no whole food from Group 1 remaining. UPFs are created with chemical modification processes that can only be carried out in industrial facilities, such as fractionation, hydrolysis, and hydrogenation. In addition, chemical additives are often included, such as artificial flavours, sweeteners, colorants, emulsifiers, and thickeners^(2)^. Examples of UPFs include sugar-sweetened soft drinks, chips and crisps, hot dogs, confectionery, and pre-prepared meals (Figure 1). These foods transcend traditional food categories due to their amalgamation of different ingredients and novel components.

**Supplementary Table 1**: Description of UPF sub-types for the NDNS data and their corresponding descriptions, NDNS sub-categories, and example food items.

| **Sub-type** | **Description** | **Corresponding NDNS Categories** | **Examples** |
| --- | --- | --- | --- |
| Alcohol | Ultra-processed wine, beer, cider, liquor, and other beverages containing alcohol | “Alcoholic Soft Drinks,” “Cider and Perry,” “Fortified Wine,” “Liqueurs,” “Spirits,” “Low Alcohol and Alcohol Free Cider & Perry” | Canned cider, vermouth, Pimm’s, rum |
| Artificial sweeteners | Sugar substitutes with little to no caloric content | “Artificial Sweeteners,” “Sugar” | Table-top sweeteners, Stevia-based sweeteners, sugar and sweetener mixes |
| Baked or roasted potatoes | Ultra-processed commercial potato products (excluding fried potatoes); homemade potatoes baked or roasted with ultra-processed ingredients (e.g. margarine) | “Other Manufactured Potato Products Fried/Baked,” “Other Fried/ Roast Potatoes Incl. Homemade Dishes,” “Other Potatoes Incl. Homemade Dishes” | Purchased frozen potatoes, potatoes roasted or mashed in margarine, potato pie with margarine short crust pastry |
| Sweet baked goods | Ultra-processed commercial biscuits, pastries, cakes and other sweet baked goods; homemade baked goods made with ultra-processed ingredients (e.g. margarine) | “Biscuits Manufactured/Retail,”  “Buns Cakes & Pastries Manufactured,” “Fruit Pies Manufactured,” “Commercial Toddlers Foods,” “Fruit Pies Homemade,” “Biscuits Homemade,” “Buns Cakes & Pastries Homemade” | Digestives, purchased apple pie, fortified baby biscuits, homemade shortbread made with margarine |
| Breakfast biscuits and cereal bars | Ultra-processed baked goods intended to be eaten for breakfast or as a snack, rather than dessert | “Biscuits Manufactured/Retail,” “Buns Cakes & Pastries Manufactured,” “Other Breakfast Cereals,” “Commercial Toddlers Foods” | Belvita Breakfast Biscuits, Kellogg’s Cereal Bars, Pop Tarts |
| Breakfast cereals | Ultra-processed commercial breakfast cereals | “High Fibre Breakfast Cereals,”  “Other Breakfast Cereals (Not High Fibre),” “Wholegrain & High Fibre Breakfast Cereals,” “Other Cereals,” “Commercial Toddler’s Foods” | Porridge, granola, Kellogs All Bran, muesli |
| Chips and fried potatoes | Ultra-processed commercial or takeaway chips and other fried potatoes; homemade potatoes fried in ultra-processed oil (e.g. oil blends) | “Chips Purchased Incl. Takeaway,”  “Other Manufactured Potato Products Fried/Baked,” “Other Fried/Roast Potatoes Incl. Homemade Dishes” | Frozen chips, McDonald’s chips, Potatoes fried in margarine |
| Chocolate confectionery | Ultra-processed sweets containing chocolate | “Chocolate Confectionery,” “Sweet Spreads Fillings And Icing” | Milk chocolate bar, chocolate sauce |
| Coated poultry and fish | Ultra-processed commercial or takeaway poultry and fish coated in batter or flour and fried; homemade equivalents fried in ultra-processed oil (e.g. oil blends) | “Manufactured Coated Chicken and Turkey Products,” “White Fish Coated Or Fried” | Purchased coated and fried chicken breast, takeaway chicken fingers, battered and fried haddock |
| Sugar confectionery | Ultra-processed sweets, not containing chocolate | “Sugar Confectionery” | Chewing gum, lollipops, marshmallows |
| Crackers and savoury biscuits | Ultra-processed baked goods that are savoury | “Biscuits Manufactured/Retail,” “Buns Cakes & Pastries Homemade,” “Commercial Toddlers Foods” | Cheese sandwich biscuits, savoury scones |
| Crisps and savoury snacks | Ultra-processed commercial crisps and other savoury foods intended to be eaten as a snack | “Crisps And Savoury Snacks,” “Nuts and Seeds,” “Other Cereals,” “Commercial Toddlers Foods” | Potato crisps, Bombay Mix, prawn crackers |
| Dairy alternatives | Ultra-processed milk, cheese, and yogurt non-dairy alternatives | “Other Milk,” “Other Cheese,” “Yogurt,” “Cream (Incl. Imitation Cream,” “Nuts and Seeds,” “Savoury Sauces Pickles Gravies and Condiments” | Lactose-free cheese, coconut milk alternative, soya yogurt alternative |
| Meal replacements and sports foods | Ultra-processed meal replacements, protein shakes, energy and electrolyte drinks, and nutrition supplements | “Beverages Dry Weight,” “Nutrition Powders And Drinks,” “Other Nutrient Supplements,” “Sugar Confectionery” | Protein drink, nutrition supplement drink, energy gels, Lucozade tablets |
| Egg dishes | Eggs cooked with ultra-processed ingredients (e.g. margarine) | “Other Eggs And Egg Dishes Incl. Homemade” | Scrambled egg in margarine |
| Fruit drinks and juices | Ultra-processed commercial beverages containing fruit or fruit-flavouring | “Fruit Juice,” “Smoothies 100% Fruit and/or Juice,” “Soft Drinks Low Calorie Concentrated/Carbonated/Still,”  “Soft Drinks Not Low Calorie Carbonated/Concentrated/Still,” “Commercial Toddlers Drinks” | Sweetened orange juice, purchased smoothie, carbonated apple juice drink |
| Hamburgers and kebabs | Ultra-processed commercial or takeaway kebab and beef, pork, chicken, or lamb burgers; homemade equivalents cooked with ultra-processed ingredients (e.g. margarine) | “Burgers And Kebabs Purchased,”  “Other Pork Incl. Homemade Recipe Dishes,” “Other Lamb Incl. Homemade Recipe Dishes,” “Manufactured Chicken Products Incl. Ready Meals” | Bacon cheeseburger, Donner Kebab, Lamb burger |
| Industrial breads | Ultra-processed commercial breads | “Brown Granary And Wheatgerm Bread,” “White Bread (Not High Fibre; Not Multiseed Bread),” “Wholemeal Bread,”  “Other Bread” | Purchased whole meal bread, rolls, chapatis, tortillas |
| Industrial desserts | Ultra-processed commercial desserts (not Incl. sweet baked goods); homemade equivalents cooked with ultra-processed ingredients (e.g. artificial sweeteners) | “Manufactured Cereal-Based Milk Puddings,” “Manufactured Fromage Frais And Dairy Desserts,” “Manufactured Sponge Puddings,” “Ice Cream,” “Manufactured Egg Products Incl. Ready Meals,” “Homemade Dairy Desserts,” “Homemade Cereal Based Puddings,” “Homemade Other Eggs And Egg Dishes,” “Homemade Sponge Puddings,” “Other Cereals,” “Sweet Spreads Fillings And Icing,” “Preserves” “Cream (Incl. Imitation Cream),” “Commercial Toddler’s Foods”  “Other Fruit Not Canned” | Purchased rice pudding, chocolate mousse, ice cream, buttercream icing, lemon curd, jelly fruit cups |
| Margarine and other spreads | Ultra-processed substitutes for butter and other spreads | “Block Margarine,” “Low Fat Spread Not Polyunsaturated,” “Polyunsaturated Low Fat Spread,” “Polyunsaturated Margarine,” “Reduced Fat Spread (Not Polyunsaturated, Polyunsaturated)”,  “Soft Margarine Not Polyunsaturated” | Reduced fat spread, margarine, olive oil spread |
| Meat alternatives | Ultra-processed meat alternatives not containing meat | “Meat Alternatives Incl. Ready Meals & Homemade Dish,” “Beans And Pulses Incl. Ready Meal & Homemade Dishes” “Other Manufactured Vegetable Products,” “Other Vegetables Incl. Homemade Dishes, | Vegetarian haggis, veggie burger, bean burger |
| Cheese and cheese spreads | Ultra-processed commercial milk-based cheeses and cheese spreads | “Other Cheese,” “Other Pasta Incl. Homemade Dishes” | Processed cheese slices, nacho cheese, macaroni cheese |
| Milk-based drinks | Ultra-processed beverages that are majority milk, includes coffee drinks with majority milk | “Beverages Dry Weight,” “Other Milk,” “Skimmed Milk,” “Cream (Incl. Imitation Cream),” “Coffee (Made-Up Weight)” | Hot chocolate, milkshake, latte |
| Yogurt | Ultra-processed commercial yogurt | “Yogurt,” “Commercial Toddler’s Food” | Yogurt, yogurt drinks |
| Mixes | Ultra-processed foods requiring additional preparation before heating and consumption, such as dilution or re-hydration | “Soup Manufactured/Retail,” “Savoury Sauces Pickles Gravies & Condiments,” “Other Cereals,” “Manufactured Other Potato Products & Dishes,” “Other Pasta Incl. Homemade Dishes,” “Other Chicken/Turkey Incl. Homemade Recipe Dishes,” “Commercial Toddler’s Foods” | Soup mixes, stock cubes, instant potatoes, casserole mix |
| Other processed meat and seafood | Meats cooked with ultra-processed ingredients, not included in other sub-types | “Homemade Meat Pies And Pastries,” “Other Meat Incl. Homemade Recipe Dishes,” “Other Pork Incl. Homemade Recipe Dishes,” “Other White Fish Incl. Homemade Dishes,” “Other Shellfish Incl. Homemade Dishes” | Lamb Cornish pastry, sweet and sour pork, fried coated squid |
| Other UPFs | Ultra-processed food items not fitting within other sub-types | “Commercial Toddler’s Drinks,” “Commercial Toddler’s Foods,” “Infant Formula,” “Other Cereals,” “Other Fruit Not Canned,” “Other Pasta Incl. Homemade Dishes,” “Other Shellfish Incl. Homemade Dishes,” “Savoury Sauces Pickles Gravies & Condiments,” “Sugar” | Yorkshire pudding, low-protein pasta, vanilla extract, low-sodium salt, golden syrup |
| Packaged pre-prepared meals | Ultra-processed commercial or takeaway foods that can be eaten immediately or after heating | “Beans And Pulses Incl. Ready Meal & Homemade Dishes,” “Manufactured Beef Products Incl. Ready Meals,” “Manufactured Canned Tuna Products Incl. Ready Meals,” “Manufactured Chicken Products Incl. Ready Meals,” “Manufactured Egg Products Incl. Ready Meals,” “Manufactured Lamb Products Incl. Ready Meals,” “Manufactured Meat Pies And Pastries,” “Manufactured Oily Fish Products Incl. Ready Meals,” “Manufactured Pork Products Incl. Ready Meals,” “Manufactured Shellfish Products Incl. Ready Meals,” “Manufactured White Fish Products Incl. Ready Meals,” “Other Manufactured Vegetable Products Incl. Ready Meals,” “Other Meat Products Manufactured  Incl. Ready Meals,” “Pasta Manufactured Products & Ready Meals,” “Ready Meals Based On Sausages, Bacon, Ham,” “Rice Manufactured Products & Ready Meals,” “Salad And Other Raw Vegetables,” “Manufactured Other Potato Products & Dishes” “Soup Manufactured/ Retail,” “Baked Beans,” “Other Cereals,” “Other Cheese,” “Commercial Toddlers Foods” | Canned soups, Tesco ready meals, takeaway ribs |
| Pizza | Ultra-processed commercial or takeaway pizza | “Pizza” | Pizza |
| Processed vegetables | Vegetables cooked with ultra-processed ingredients (e.g. margarine), not included in other sub-types | “Other Vegetables Incl. Homemade Dishes,” “Tomatoes Not Raw,” “Nuts And Seeds” | Tomatoes fried in margarine |
| Reconstituted meat products | Ultra-processed commercial meat products made through the industrial separation and recombination of meat; homemade dishes containing reconstituted meat products | “Homemade Meat Pies and Pastries,” “Liver And Dishes,” “Manufactured Beef Products Incl. Ready Meals,” “Manufactured Chicken Products Incl. Ready Meals,” “Manufactured Pork Products Incl. Ready Meals,” “Manufactured Shellfish Products Incl. Ready Meals,” “Other Meat Products Manufactured Incl. Ready Meals,” “Other Bacon And Ham Incl. Homemade Dishes,” “Other Meat Incl. Homemade Recipe Dishes,” “Other Pasta Incl. Homemade Dishes,” “Other Sausages Incl. Homemade Dishes,” “Soup Homemade” | Corned beef pie, turkey roll, liver pate, meatballs, crabsticks, homemade soup with reconstituted ham |
| Sauces, dressings, and gravies | Ultra-processed commercial sauces, dressings, and gravies (Incl. condiments and preserves); homemade meals cooked with these sauces, dressings, and gravies | “Savoury Sauces Pickles Gravies & Condiments,” “Preserves,” “Manufactured Beef Products Incl. Ready Meals,” “Manufactured Egg Products Incl. Ready Meals,” “Manufactured Shellfish Products Incl. Ready Meals,” “Other Beef & Veal Incl. Homemade Recipe Dishes,” “Other Chicken/Turkey Incl. Homemade Recipe Dishes,” “Other Lamb Incl. Homemade Recipe Dishes,” “Other Pasta Incl. Homemade Dishes,” “Other Vegetables Incl. Homemade Dishes,” “Pasta Manufactured Products & Ready Meals,” “Commercial Toddlers Foods” | Chicken curry made with purchased sauce, marmalade, French dressing, gravy |
| Soft drinks | Ultra-processed non-alcoholic beverages, not containing fruit or fruit-flavour | “Soft Drinks Not Low Calorie Still/Carbonated/ Concentrated,” “Soft Drinks Low Calorie Still/Carbonated/Concentrated,” “Beverages Dry Weight,” “Bottled Water Still Or Carbonated” | Coca Cola, flavoured water |
| Tea and coffee | Ultra-processed commercial beverages containing tea and coffee or their substitutes | “Tea (Made-Up Weight),” “Coffee (Made-Up Weight),” “Beverages Dry Weight” | Instant tea, coffee substitutes (i.e. chicory powder) |

Abbreviations: Incl. = including.

**Supplementary Table 2:** Sociodemographic characteristics of adolescents from years 1-11 of the NDNS study with missing data for at least one variable of interest (n=71).

| **Sociodemographic Characteristic**^†^ | | **Weighted %N (95% CI)*** |
| --- | --- | --- |
| **Age** | | |
|  | 11 | 13.8 (6.1, 28.1) |
|  | 12 | 2.5 (0.4, 12.8) |
|  | 13 | 8.6 (3.3, 20.8) |
|  | 14 | 2.2 (0.8, 6.1) |
|  | 15 | 18.8 (9.5, 33.8) |
|  | 16 | 20.3 (11.5, 33.2) |
|  | 17 | 7.5 (3.3, 15.9) |
|  | 18 | 26.4 (14.5, 43.1) |
| **Sex** | | |
|  | Female | 46.3 (32.2, 61.1) |
|  | Male | 53.7 (38.9, 67.8) |
| **Ethnicity** | | |
|  | White | 64.9 (49.2, 77.9) |
|  | Asian or Asian British | 7.3 (2.4, 19.9) |
|  | Black or Black British | 11.9 (4.5, 28.1) |
|  | Mixed ethnic group | 7.0 (2.0, 21.6) |
|  | Any other group | 5.7 (2.0, 15.6) |
|  | *Data Missing* | *3.1 (0.5, 16.4)* |
| **Region** | | |
|  | England: North | 12.2 (5.5, 24.8) |
|  | England: Central/Midlands | 29.2 (17.5, 44.7) |
|  | England: South (incl. London) | 40.0 (26.3, 55.4) |
|  | Northern Ireland | 2.4 (1.0, 5.3) |
|  | Scotland | 11.5 (5.1, 24.1) |
|  | Wales | 4.7 (2.0, 10.6) |
| **Parental Occupation** | | |
|  | Higher managerial, administrative and professional occupations | 10.4 (3.7, 25.9) |
|  | Intermediate occupations | 4.6 (1.2, 16.7) |
|  | Routine and manual occupations | 7.7 (3.0, 18.4) |
|  | Never worked | 0.0 (0.0, 0.0) |
|  | *Data Missing* | *77.3 (62.1, 87.6)* |
| **Housing Tenure** | | |
|  | Own outright | 13.4 (6.5, 25.5) |
|  | Own with mortgage | 20.8 (11.6, 34.5) |
|  | Rent privately | 23.3 (11.9, 40.5) |
|  | Rent social housing | 17.2 (9.1, 30.0) |
|  | *Data Missing* | *25.3 (14.6, 40.2)* |

*Weighted percentage of sample (%N) and 95% confidence interval (CI) are reported. Percentages and means are weighed based on non-selection and non-response survey weights provided by NDNS year 2008-2019.

^†^ “Missing” indicates those within the sample of excluded participants that were missing data for that characteristic.

**Supplementary Table 3:** Average daily relative energy from UPFs (% kcal/day) and weight from UPFs (% g/day) in adolescents (11-18 years old) from years 1-11 of the NDNS study for the overall sample (n=3199) and each sociodemographic subgroup.

|  | | **UPF Relative Diet Contribution** | |
| --- | --- | --- | --- |
| **Sociodemographic Characteristic** | | **% kcal/day (95% CI)^*^** | **% g/day (95% CI)^*^** |
| **Overall Sample** | | 65.8 (65.1, 66.4) | 44.6 (43.7, 45.5) |
| **Age** | | |  |
|  | 11 | 65.4 (63.8, 67.0) | 45.1 (42.8, 47.4) |
|  | 12 | 66.1 (64.5, 67.8) | 45.1 (42.9, 47.4) |
|  | 13 | 67.6 (65.9, 69.4) | 47.1 (44.8, 49.4) |
|  | 14 | 66.6 (64.9, 68.3) | 45.2 (42.7, 47.7) |
|  | 15 | 66.0 (64.4, 67.6) | 45.5 (42.9, 48.0) |
|  | 16 | 66.0 (64.5, 67.5) | 42.4 (40.2, 44.7) |
|  | 17 | 64.4 (62.3, 66.5) | 45.0 (42.1, 47.8) |
|  | 18 | 63.6 (61.6, 65.6) | 40.9 (38.4, 43.4) |
| **Sex** | | |  |
|  | Female | 65.7 (64.8, 66.5) | 43.4 (42.2, 44.6) |
|  | Male | 65.9 (65.0, 66.7) | 45.7 (44.4, 46.9) |
| **Ethnicity** | | |  |
|  | White | 67.3 (66.6, 67.9) | 46.5 (45.6, 47.5) |
|  | Asian or Asian British | 57.3 (55.0, 59.6) | 32.9 (30.3, 35.4) |
|  | Black or Black British | 60.3 (57.6, 63.1) | 38.9 (35.3, 42.5) |
|  | Mixed ethnic group | 63.4 (60.2, 66.6) | 44.4 (39.2, 49.5) |
|  | Any other group | 56.8 (50.3, 63.4) | 29.7 (25.6, 33.8) |
| **Parental Occupation** | | |  |
|  | Higher managerial, administrative and professional occupations | 63.8 (62.9, 64.7) | 42.1 (40.8, 43.4) |
|  | Intermediate occupations | 65.8 (64.5, 67.1) | 43.7 (41.9, 45.6) |
|  | Routine and manual occupations | 68.2 (67.1, 69.3) | 48.4 (46.8, 50.0) |
|  | Never worked | 65.5 (62.1, 68.9) | 42.6 (38.0, 47.2) |
| **Housing Tenure** | | |  |
|  | Own outright | 61.5 (59.8, 63.1) | 39.3 (37.0, 41.7) |
|  | Own with mortgage | 65.5 (64.7, 66.3) | 44.2 (43.0, 45.3) |
|  | Rent privately | 65.8 (64.0, 67.6) | 43.7 (41.3, 46.2) |
|  | Rent social housing | 69.0 (67.5, 70.5) | 49.2 (47.3, 51.2) |
| **Region** | | |  |
|  | England: North | 67.3 (66.1, 68.5) | 47.6 (45.8, 49.3) |
|  | England: Central/Midlands | 67.0 (65.7, 68.4) | 47.1 (45.1, 49.2) |
|  | England: South (incl. London) | 63.8 (62.8, 64.9) | 41.4 (39.9, 42.8) |
|  | Northern Ireland | 67.8 (66.6, 69.0) | 44.6 (42.8, 46.5) |
|  | Scotland | 67.1 (64.9, 69.3) | 46.6 (43.6, 49.6) |
|  | Wales | 67.6 (66.1, 69.2) | 46.5 (44.1, 48.9) |

Incl., including

*Weighted average value and 95% confidence interval (95% CI).

**Supplementary Table 4:** Average daily relative energy from all UPF sub-types (% kcal/day) in adolescents (11-18 years old) from years 1-11 of the NDNS study (n=3199).

| **UPF Sub-Type** | **Relative Energy**  **% kcal/day (95% CI)*** |
| --- | --- |
| Industrial breads | 11.9 (11.6, 12.2) |
| Sweet baked goods | 6.8 (6.4, 7.1) |
| Packaged pre-prepared meals | 5.4 (5.1, 5.6) |
| Breakfast cereals | 4.1 (3.9, 4.3) |
| Crisps and savoury snacks | 3.9 (3.7, 4.1) |
| Chips and fried potatoes | 3.9 (3.6, 4.1) |
| Pizza | 3.8 (3.5, 4.1) |
| Chocolate confectionery | 3.1 (2.9, 3.3) |
| Soft drinks | 2.9 (2.7, 3.1) |
| Reconstituted meat products | 2.7 (2.6, 2.9) |
| Sauces, dressings, and gravies | 2.5 (2.4, 2.7) |
| Coated poultry and fish | 2.5 (2.3, 2.6) |
| Industrial desserts | 2.4 (2.2, 2.6) |
| Margarine and other spreads | 1.7 (1.6, 1.8) |
| Fruit drinks and juices | 1.5 (1.4, 1.6) |
| Hamburgers and kebabs | 1.3 (1.1, 1.4) |
| Milk-based drinks | 1.1 (1.0, 1.2) |
| Sugar confectionery | 1.0 (0.9, 1.1) |
| Yogurt | 0.8 (0.7, 0.8) |
| Breakfast biscuits and cereal bars | 0.6 (0.5, 0.7) |
| Baked or roasted potatoes | 0.5 (0.4, 0.5) |
| Crackers and savoury biscuits | 0.4 (0.3, 0.4) |
| Alcohol | 0.3 (0.2, 0.3) |
| Cheese and cheese spreads | 0.2 (0.2, 0.3) |
| Meat alternatives | 0.2 (0.1, 0.2) |
| Other processed meat and seafood | 0.1 (0.1, 0.2) |
| Meal replacements and sports foods | 0.1 (0.1, 0.2) |
| Other UPFs | 0.1 (0.1, 0.2) |
| Dairy alternatives | 0.1 (0.1, 0.2) |
| Mixes | 0.1 (0.1, 0.1) |
| Egg dishes | 0.0 (0.0, 0.0) |
| Tea and coffee | 0.0 (0.0, 0.0) |
| Processed vegetables | 0.0 (0.0, 0.0) |
| Artificial sweeteners | 0.0 (0.0, 0.0) |

*Weighted average values and 95% confidence interval (CI) are reported.

**Supplementary Table 5:** Average daily relative weight from all UPF sub-types (% g/day) in adolescents (11-18 years old) from years 1-11 of the NDNS study (n=3199).

| **UPF Sub-Type** | **Relative Weight**  **% g/day (95% CI)*** |
| --- | --- |
| Soft drinks | 16.4 (15.7, 17.0) |
| Fruit drinks and juices | 4.5 (4.2, 4.7) |
| Industrial breads | 4.3 (4.1, 4.4) |
| Packaged pre-prepared meals | 3.3 (3.1, 3.4) |
| Chips and fried potatoes | 1.6 (1.5, 1.7) |
| Sauces, dressings, and gravies | 1.6 (1.5, 1.7) |
| Sweet baked goods | 1.6 (1.5, 1.6) |
| Pizza | 1.4 (1.3, 1.5) |
| Reconstituted meat products | 1.2 (1.1, 1.3) |
| Industrial desserts | 1.1 (1.0, 1.2) |
| Breakfast cereals | 1.1 (1.0, 1.1) |
| Milk-based drinks | 0.9 (0.8, 1.1) |
| Coated poultry and fish | 0.9 (0.9, 1.0) |
| Crisps and savoury snacks | 0.9 (0.8, 0.9) |
| Yogurt | 0.7 (0.7, 0.8) |
| Chocolate confectionery | 0.6 (0.5, 0.6) |
| Hamburgers and kebabs | 0.5 (0.4, 0.5) |
| Sugar confectionery | 0.3 (0.3, 0.4) |
| Alcohol | 0.3 (0.2, 0.4) |
| Baked or roasted potatoes | 0.3 (0.3, 0.3) |
| Margarine and other spreads | 0.3 (0.3, 0.3) |
| Mixes | 0.2 (0.1, 0.2) |
| Dairy alternatives | 0.2 (0.1, 0.2) |
| Breakfast biscuits and cereal bars | 0.1 (0.1, 0.2) |
| Meat alternatives | 0.1 (0.1, 0.1) |
| Cheese and cheese spreads | 0.1 (0.1, 0.1) |
| Crackers and savoury biscuits | 0.1 (0.1, 0.1) |
| Meal replacements and sports foods | 0.1 (0.0, 0.01) |
| Other processed meat and seafood | 0.0 (0.0, 0.01) |
| Other UPFs | 0.0 (0.0, 0.0) |
| Tea and coffee | 0.0 (0.0, 0.1) |
| Egg dishes | 0.0 (0.0, 0.0) |
| Processed vegetables | 0.0 (0.0, 0.0) |
| Artificial sweeteners | 0.0 (0.0, 0.0) |

*Weighted average values and 95% confidence interval (CI) are reported.

**Supplementary Table 6**: Description of most informative PCs detected through PCA of daily relative energy UPF sub-type intake data for adolescents (11-18 years old) from years 1-11 of the NDNS study (n=3199).

|  | **Factor Loading Coefficient*** | | |
| --- | --- | --- | --- |
| **UPF Sub-Type**^†^ | **PC 1** | **PC 2** | **PC 3** |
| Alcohol | 0.1834 | -0.0688 | 0.2427 |
| Artificial sweeteners | -0.0620 | 0.0260 | 0.2626 |
| Sweet baked goods | -0.2031 | -0.2991 | -0.3216 |
| Breakfast cereals | -0.2527 | -0.3678 | 0.1735 |
| Cheese and cheese spreads | -0.0795 | 0.0106 | 0.2611 |
| Chips and fried potatoes | 0.6753 | 0.1397 | -0.0328 |
| Chocolate confectionery | -0.0082 | -0.1140 | -0.2391 |
| Coated poultry and fish | 0.4257 | 0.1785 | -0.1659 |
| Fruit drinks and juices | 0.0385 | 0.0326 | -0.2260 |
| Hamburgers and kebabs | 0.5153 | 0.0005 | -0.0087 |
| Industrial breads | -0.3629 | 0.7224 | -0.0100 |
| Industrial desserts | -0.1508 | -0.1329 | -0.3475 |
| Margarine and other spreads | -0.2258 | 0.6632 | -0.0596 |
| Meat alternatives | -0.2317 | -0.1482 | 0.1758 |
| Mixes | -0.1044 | 0.0813 | 0.2393 |
| Pizza | 0.1119 | -0.2031 | 0.3343 |
| Reconstituted meat products | 0.0470 | 0.2288 | -0.3271 |
| Sauces, dressings, and gravies | -0.0066 | 0.0926 | 0.4081 |
| Soft drinks | 0.5726 | 0.0237 | 0.0815 |
| Sugar confectionery | 0.0762 | -0.1399 | -0.3609 |
| Tea and coffee | 0.0532 | 0.1348 | 0.2210 |
| Yogurt | -0.2524 | -0.2261 | -0.0837 |

*Factor loadings for each of three informative principle components (PCs) for UPF sub-type relative energy data: PC 1, PC 2, and PC 3.

^†^ Baked and roasted potatoes; other processed meat; processed vegetables; meal replacements and sports food; breakfast biscuits and cereal bars; egg dishes; crisps and savoury snacks; packaged pre-prepared meals; milk-based drinks; dairy alternatives; crackers and savoury biscuits; and other UPF sub-categories were excluded based on low factor loadings in previous round of PCA (factor loading < 0.20).

**Supplementary Table 7**: Description of most informative PCs detected through PCA of daily relative weight UPF sub-type intake data for adolescents (11-18 years old) from years 1-11 of the NDNS study (n=3199).

|  | **Factor Loading Coefficient*** | | | |
| --- | --- | --- | --- | --- |
| **UPF Sub-Type** | **PC 1** | **PC 2** | **PC 3** | **PC 4** |
| Sweet baked goods | 0.0976 | 0.1744 | 0.4247 | -0.0165 |
| Breakfast cereals | -0.1909 | 0.1710 | 0.2083 | 0.2785 |
| Chips and fried potatoes | 0.5466 | -0.3315 | -0.0800 | 0.3419 |
| Chocolate confectionery | 0.2335 | 0.0812 | 0.2757 | -0.3180 |
| Coated poultry and fish | 0.4330 | -0.1812 | -0.0847 | 0.2854 |
| Crackers and savoury biscuits | -0.0903 | 0.1954 | 0.2803 | 0.1662 |
| Crisps and savoury snacks | 0.2976 | -0.0138 | 0.1095 | -0.3623 |
| Dairy alternatives | -0.2661 | -0.0632 | -0.0849 | -0.0438 |
| Fruit drinks and juices | 0.2974 | -0.0871 | 0.2338 | -0.1977 |
| Hamburgers and kebabs | 0.3746 | -0.3676 | 0.0404 | 0.3408 |
| Industrial breads | 0.3631 | 0.6829 | -0.2082 | 0.1069 |
| Industrial desserts | 0.0992 | 0.1970 | 0.4116 | 0.0331 |
| Margarine and other spreads | 0.3941 | 0.6010 | -0.2043 | 0.0800 |
| Meat alternatives | -0.2661 | 0.0730 | 0.0042 | -0.0397 |
| Milk-based drinks | -0.0731 | -0.0976 | 0.2665 | 0.4878 |
| Mixes | -0.0856 | 0.1002 | -0.3162 | -0.0118 |
| Other UPFs | -0.1047 | 0.1042 | 0.2369 | -0.1258 |
| Packaged pre-prepared meals | 0.2174 | 0.0786 | -0.0425 | -0.1708 |
| Reconstituted meat products | 0.3801 | 0.3135 | 0.0124 | 0.1107 |
| Soft drinks | 0.4661 | -0.3365 | 0.0291 | -0.2953 |
| Sugar confectionery | 0.2220 | -0.0349 | 0.4258 | -0.1022 |
| Tea and coffee | 0.0355 | -0.0277 | -0.2978 | 0.0609 |
| Yogurt | -0.1764 | 0.1043 | 0.3372 | 0.2754 |

*Factor loadings for each of four informative principle components (PCs) for UPF sub-type relative weight data: PC 1, PC 2, PC 3, and PC 4.

^†^ Baked and roasted potatoes; other processed meat; processed vegetables; dietary meal replacements and sports food; breakfast biscuits and cereal bars; egg dishes; sauces, dressings, and gravies; pizza; cheese and cheese spreads; alcohol; and artificial sweeteners sub-categories were excluded based on low factor loadings in previous round of PCA (factor loading < 0.20).

**Supplementary Figure 1:** Scree plots describing PCA results. Plot of the percentage of the explained variance in the data for each of the principal components (“Dimensions”) detected in data describing adolescents’ daily relative energy from UPF sub-types (A) and daily relative weight from UPF sub-types (B).


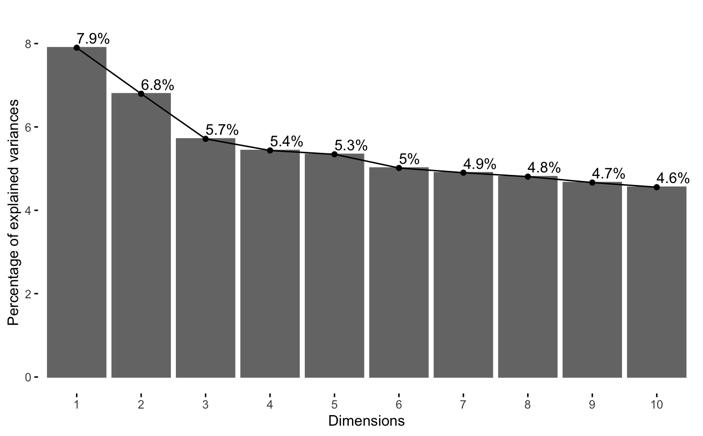

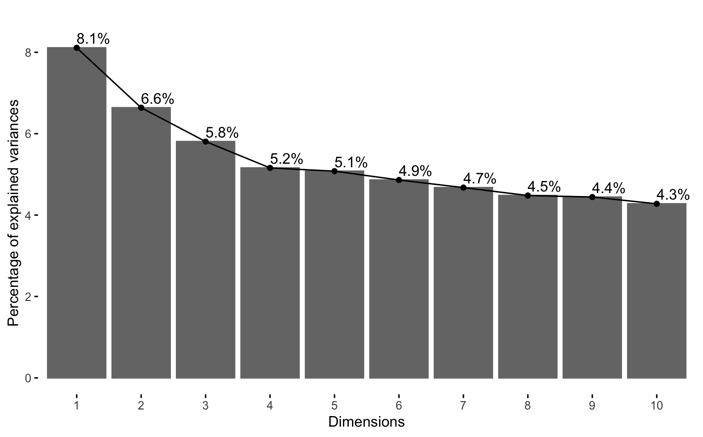


**A**

**B**

**Supplementary Table 8:** Average daily relative weight from each of the UPF sub-types (% g/day) in adolescents (11-18 years old) from years 1-11 of the NDNS study for the overall sample (n=3199) and each identified dietary pattern (using PCA and cluster analysis).

|  | **Relative Weight (% g/day) (95% CI)^*^** | | | |
| --- | --- | --- | --- | --- |
| **UPF Sub-Type** | **Overall Sample**  (n=3199) | **Restrictive**  (n=1618)^†^ | **Traditional**  (n=572)^†^ | **Permissive**  (n=1009)^†^ |
| Alcohol | 0.3 (0.2, 0.4) | 0.3 (0.2, 0.4) | 0.2 (0.0, 0.4) | 0.4 (0.2, 0.6) |
| Artificial sweeteners | 0.0 (0.0, 0.0) | 0.0 (0.0, 0.0) | 0.0 (0.0, 0.0) | 0.0 (0.0, 0.0) |
| Baked or roasted potatoes | 0.3 (0.3, 0.4) | 0.3 (0.2, 0.3) | 0.4 (0.3, 0.6) | 0.4 (0.3, 0.4) |
| Sweet baked goods | 1.6 (1.5, 1.6) | 1.5 (1.4, 1.6) | **2.0 (1.7, 2.2)** | 1.5 (1.3, 1.6) |
| Breakfast biscuits and cereal bars | 0.1 (0.1, 0.2) | 0.2 (0.1, 0.2) | 0.1 (0.1, 0.2) | 0.1 (0.1, 0.2) |
| Breakfast cereals | 1.1 (1.0, 1.1) | **1.2 (1.1, 1.3)** | 1.2 (1.0, 1.4) | **0.7 (0.7, 0.8)** |
| Cheese and cheese spreads | 0.1 (0.1, 0.1) | 0.1 (0.1, 0.1) | 0.1 (0.1, 0.1) | 0.1 (0.1, 0.1) |
| Chips and fried potatoes | 1.6 (1.5, 1.7) | **0.9 (0.8, 1.0)** | 1.7 (1.5, 1.9) | **2.8 (2.6, 3.0)** |
| Chocolate confectionery | 0.6 (0.6, 0.6) | **0.4 (0.4, 0.5)** | 0.7 (0.6, 0.8) | **0.8 (0.7, 0.9)** |
| Coated poultry and fish | 0.9 (0.9, 1.0) | **0.5 (0.4, 0.6)** | 1.2 (1.0, 1.4) | **1.6 (1.5, 1.8)** |
| Crackers and savoury biscuits | 0.1 (0.1, 0.1) | 0.1 (0.1, 0.1) | 0.1 (0.1, 0.2) | **0.0 (0.0, 0.1)** |
| Crisps and savoury snacks | 0.9 (0.8, 0.9) | **0.6 (0.6, 0.7)** | 1.0 (0.8, 1.10) | **1.3 (1.1, 1.5)** |
| Dairy alternatives | 0.2 (0.1, 0.3) | 0.3 (0.2, 0.4) | **0.0 (0.0, 0.1)** | **0.0 (0.0, 0.1)** |
| Meal replacements  and sports foods | 0.1 (0.0, 0.1) | 0.1 (0.0, 0.1) | **0.0 (0.0, 0.0)** | 0.0 (0.0, 0.0) |
| Egg dishes | 0.0 (0.0, 0.0) | 0.0 (0.0, 0.0) | 0.0 (0.0, 0.0) | 0.0 (0.0, 0.0) |
| Fruit drinks and juices | 4.5 (4.2, 4.7) | **3.0 (2.8, 3.3)** | 4.5 (3.8, 5.2) | **7.0 (6.4, 7.5)** |
| Hamburgers and kebabs | 0.5 (0.4, 0.5) | **0.2 (0.2, 0.2)** | **0.3 (0.2, 0.4)** | **1.1 (1.0, 1.3)** |
| Industrial breads | 4.3 (4.1, 4.4) | **3.5 (3.3, 3.6)** | **7.9 (7.6, 8.3)** | **3.5 (3.4, 3.7)** |
| Industrial desserts | 1.1 (1.0, 1.1) | 1.0 (0.9, 1.1) | **1.6 (1.4, 1.8)** | 1.1 (0.9, 1.2) |
| Margarine and other spreads | 0.3 (0.3, 0.3) | **0.2 (0.2, 0.2)** | **0.8 (0.8, 0.9)** | **0.2 (0.2, 0.3)** |
| Meat alternatives | 0.1 (0.1, 0.1) | **0.2 (0.1, 0.2)** | **0.0 (0.0, 0.0)** | **0.0 (0.0, 0.0)** |
| Milk-based drinks | 0.9 (0.8, 1.1) | 1.1 (0.9, 1.3) | **0.6 (0.4, 0.7)** | 0.9 (0.7, 1.1) |
| Mixes | 0.2 (0.2, 0.2) | 0.2 (0.2, 0.3) | 0.3 (0.1, 0.4) | **0.1 (0.1, 0.1)** |
| Other processed meat and seafood | 0.1 (0.0, 0.1) | 0.1 (0.0, 0.1) | 0.1 (0.0, 0.1) | 0.1 (0.0, 0.1) |
| Other UPFs | 0.0 (0.0, 0.1) | 0.1 (0.0, 0.1) | 0.0 (0.0, 0.0) | 0.0 (0.0, 0.0) |
| Packaged pre-prepared meals | 3.3 (3.1, 3.4) | **2.7 (2.5, 2.8)** | **4.1 (3.6, 4.6)** | **3.9 (3.6, 4.3)** |
| Pizza | 1.4 (1.3, 1.5) | 1.3 (1.1, 1.4) | 1.4 (1.1, 1.7) | 1.6 (1.4, 1.8) |
| Processed vegetables | 0.0 (0.0, 0.0) | 0.0 (0.0, 0.0) | 0.0 (0.0, 0.0) | 0.0 (0.0, 0.0) |
| Reconstituted meat products | 1.2 (1.1, 1.3) | **0.8 (0.7, 0.8)** | **2.4 (2.1, 2.6)** | 1.3 (1.2, 1.4) |
| Sauces, dressings, and gravies | 1.6 (1.5, 1.7) | 1.5 (1.4, 1.6) | 1.8 (1.6, 2.1) | 1.7 (1.5, 1.8) |
| Soft drinks | 16.4 (15.7, 17.0) | **10.8 (10.2, 11.5)** | **13.8 (12.5, 15.0)** | **28.3 (27.1, 29.6)** |
| Sugar confectionery | 0.3 (0.3, 0.4) | **0.2 (0.2, 0.2)** | 0.3 (0.2, 0.3) | **0.6 (0.5, 0.7)** |
| Tea and coffee | 0.0 (0.0, 0.1) | 0.0 (0.0, 0.0) | 0.1 (0.0, 0.2) | 0.0 (0.0, 0.0) |
| Yogurt | 0.7 (0.7, 0.8) | **0.9 (0.8, 1.1)** | 0.7 (0.5, 0.9) | **0.4 (0.3, 0.5)** |

*Weighted average values and 95% confidence interval (CI) are reported.

^†^ Values highlighted blue are significantly lower than the sample average. Values highlighted yellow are significantly higher than the sample average.

**Supplementary Table 9:** Average daily relative energy from each of the UPF sub-types (% kcal/day) in adolescents (11-18 years old) from years 1-11 of the NDNS study for the overall sample (n=3199) and each identified dietary pattern (using PCA and cluster analysis).

|  | **Relative Energy (%kcal/day) (95% CI)*** | | | | | | | |
| --- | --- | --- | --- | --- | --- | --- | --- | --- |
| **UPF Sub-Type** | **Full Sample**  (n = 3199) | | **Restrictive**^†^  (n =1 ,542) | | **Traditional**^†^  (n = 823) | | **Permissive**^†^  (n =834) | |
| Alcohol | | 0.3 (0.2, 0.3) | | **0.1 (0.1, 0.1)** | | **0.0 (0.0, 0.1)** | | **0.8 (0.6, 1.1)** |
| Artificial sweeteners | | 0.0 (0.0, 0.0) | | 0.0 (0.0, 0.0) | | 0.0 (0.0, 0.0) | | 0.0 (0.0, 0.0) |
| Baked or roasted potatoes | | 0.5 (0.4, 0.5) | | 0.5 (0.4, 0.6) | | 0.4 (0.3, 0.5) | | 0.5 (0.4, 0.7) |
| Sweet baked goods | | 6.8 (6.4, 7.1) | | **8.8 (8.3, 9.3)** | | **4.8 (4.3, 5.3)** | | **4.7 (4.2, 5.1)** |
| Breakfast biscuits and cereal bars | | 0.6 (0.5, 0.7) | | 0.7 (0.6, 0.8) | | 0.7 (0.5, 0.9) | | **0.4 (0.3, 0.5*)*** |
| Breakfast cereals | | 4.1 (3.9, 4.3) | | **5.7 (5.4, 6.0)** | | **2.6 (2.3, 2.9)** | | **2.5 (2.2, 2.8)** |
| Cheese and cheese spreads | | 0.2 (0.2, 0.3) | | 0.3 (0.2, 0.3) | | 0.3 (0.2, 0.3) | | 0.2 (0.1, 0.2) |
| Chips and fried potatoes | | 3.9 (3.6, 4.1) | | **2.1 (2.0, 2.3)** | | **3.2 (2.8, 3.5)** | | **8.0 (7.5, 8.5)** |
| Chocolate confectionery | | 3.1 (2.9, 3.3) | | 3.5 (3.2, 3.8) | | **2.5 (2.2, 2.8)** | | 2.9 (2.5, 3.2) |
| Coated poultry and fish | | 2.5 (2.3, 2.6) | | **1.4 (1.2, 1.5)** | | 2.5 (2.1, 2.9) | | **4.7 (4.2, 5.1)** |
| Crackers and savoury biscuits | | 0.4 (0.3, 0.4) | | 0.5 (0.4, 0.5) | | 0.3 (0.2, 0.4) | | 0.2 (0.2, 0.3) |
| Crisps and savoury snacks | | 3.9 (3.7, 4.1) | | 3.7 (3.4, 4.0) | | 4.0 (3.6, 4.4) | | 4.2 (3.7, 4.6) |
| Dairy alternatives | | 0.1 (0.1, 0.2) | | 0.2 (0.1, 0.3) | | 0.0 (0.0, 0.1) | | 0.0 (0.0, 0.1) |
| Dietary supplements, meal replacements and sports foods | | 0.1 (0.1, 0.2) | | 0.2 (0.1, 0.3) | | 0.1 (0.0, 0.1) | | 0.1 (0.0, 0.1) |
| Egg dishes | | 0.0 (0.0, 0.0) | | 0.0 (0.0, 0.0) | | 0.0 (0.0, 0.1) | | 0.0 (0.0, 0.0) |
| Fruit drinks and juices | | 1.5 (1.4, 1.6) | | 1.4 (1.3, 1.5) | | 1.5 (1.3, 1.7) | | 1.6 (1.4, 1.8) |
| Hamburgers and kebabs | | 1.3 (1.1, 1.4) | | **0.5 (0.4, 0.6)** | | **0.7 (0.5, 0.8)** | | **3.5 (3.1, 4.0)** |
| Industrial breads | | 11.9 (11.6, 12.2) | | **9.8 (9.5, 10.2)** | | **19.0 (18.4, 19.6)** | | **8.8 (8.4, 9.3)** |
| Industrial desserts | | 2.4 (2.2, 2.6) | | **2.9 (2.7, 3.2)** | | 2.0 (1.7, 2.3) | | **1.7 (1.4, 2.0)** |
| Margarine and other spreads | | 1.7 (1.6, 1.8) | | **1.0 (0.9, 1.1)** | | **3.7 (3.5, 4.0)** | | **1.1 (1.0, 1.2)** |
| Meat alternatives | | 0.2 (0.1, 0.2) | | 0.3 (0.2, 0.3) | | 0.1 (0.0, 0.2) | | **0.0 (0.0, 0.1)** |
| Milk-based drinks | | 1.1 (1.0, 1.2) | | 1.2 (1.0, 1.4) | | **0.8 (0.6, 1.0)** | | 1.2 (1.0, 1.5) |
| Mixes | | 0.1 (0.1, 0.1) | | 0.1 (0.1, 0.1) | | **0.2 (0.1, 0.2)** | | 0.1 (0.0, 0.1) |
| Other processed meat and seafood | | 0.1 (0.1, 0.2) | | 0.1 (0.1, 0.2) | | 0.1 (0.0, 0.1) | | 0.1 (0.1, 0.2) |
| Other UPFs | | 0.1 (0.1, 0.2) | | 0.2 (0.1, 0.2) | | 0.1 (0.0, 0.1) | | 0.1 (0.0, 0.1) |
| Packaged pre-prepared meals | | 5.4 (5.1, 5.6) | | 5.3 (4.9, 5.7) | | 5.0 (4.5, 5.5) | | 5.9 (5.3, 6.5) |
| Pizza | | 3.8 (3.5, 4.1) | | 4.0 (3.6, 4.4) | | **2.3 (1.9, 2.7)** | | **4.7 (4.0, 5.4)** |
| Processed vegetables | | 0.0 (0.0, 0.0) | | 0.0 (0.0, 0.0) | | 0.0 (0.0, 0.0) | | 0.0 (0.0, 0.0) |
| Reconstituted meat products | | 2.7 (2.6, 2.9) | | **2.2 (2.0, 2.4)** | | **3.7 (3.3, 4.1)** | | 2.7 (2.4, 3.1) |
| Sauces, dressings, and gravies | | 2.5 (2.4, 2.7) | | **2.3 (2.2, 2.5)** | | 2.7 (2.5, 3.0) | | 2.8 (2.5, 3.0) |
| Soft drinks | | 2.9 (2.7, 3.1) | | **1.7 (1.5, 1.8)** | | **1.8 (1.6, 2.1)** | | **6.5 (6.0, 7.0)** |
| Sugar confectionery | | 1.0 (0.9, 1.1) | | 1.2 (1.0, 1.3) | | **0.7 (0.5, 0.8)** | | 1.1 (0.8, 1.2) |
| Tea and coffee | | 0.0 (0.0, 0.0) | | 0.0 (0.0, 0.0) | | 0.0 (0.0, 0.1) | | 0.0 (0.0, 0.0) |
| Yogurt | | 0.8 (0.7, 0.8) | | **1.2 (1.0, 1.3)** | | **0.4 (0.3, 0.5)** | | **0.3 (0.2, 0.4)** |

*Weighted average values and 95% confidence interval (95% CI) are reported.

^†^ Values highlighted blue are significantly lower than the sample average. Values highlighted yellow are significantly higher than the sample average.

**Supplementary Table 10:** Sociodemographic characteristics of adolescents (11-18 years old) from years 1-11 of the NDNS study for the overall sample (n=3199) and each identified dietary pattern (based on PCA and cluster analysis) using average daily relative energy from each of the UPF sub-types (% kcal/day).

|  |  | **Full Sample** | **Restrictive** | | **Traditional** | | | **Permissive** | | |
| --- | --- | --- | --- | --- | --- | --- | --- | --- | --- | --- |
| **Characteristic** | | **%N (95%CI)*** | **%N (95%CI)*** | **p-value**^†^ | **%N (95%CI)*** | **p-value**^†^ | | **%N (95%CI)*** | | **p-value**^†^ |
| **Age** | |  |  | <0.001 |  | 0.16 |  | | <0.001 | |
|  | 11 | 12.3 (10.9, 13.9) | 15.0 (12.9, 17.4) |  | 12.7 (9.8, 16.2) |  | | 6.6 (4.7, 9.1) | |  |
|  | 12 | 12.7 (11.3, 14.3) | 13.5 (11.5, 15.9) |  | 14.1 (11.2, 17.7) |  |  | 9.6 (7.2, 12.8) | |  |
|  | 13 | 12.5 (11.1, 14.1) | 13.2 (11.2, 15.5) |  | 15.1 (12.1, 187) |  |  | 8.5 (6.4, 11.3) | |  |
|  | 14 | 13.0 (11.6, 14.6) | 14.1 (12.0, 16.4) |  | 12.8 (10.1, 16.0) |  |  | 11.1 (8.5, 14.2) | |  |
|  | 15 | 11.0 (9.8, 12.5) | 11.3 (9.5, 13.4) |  | 11.4 (9.0, 14.4) |  |  | 10.2 (7.9, 13.0) | |  |
|  | 16 | 14.7 (13.1, 16.5) | 14.0 (11.8, 16.6) |  | 11.7 (9.1, 14.9) |  |  | 19.2 (15.7, 23.1) | |  |
|  | 17 | 13.9 (12.4, 15.6) | 10.6 (8.7, 12.8) |  | 13.8 (11.0, 17.3) |  |  | 20.7 (17.1, 24.8) | |  |
|  | 18 | 9.8 (8.5, 11.1) | 8.3 (6.7, 10.1) |  | 8.39 (6.3, 11.1) |  |  | 14.2 (11.4, 17.6) | |  |
| **Sex** | | | | | | | | | | |
|  | Female | 48.7 (46.5, 51.0) | 48.7 (45.5, 51.9) |  | 47.2 (42.7, 51.7) |  | | 50.3 (45.8, 54.9) | |  |
|  | Male | 51.3 (49.0, 53.5) | 51.3 (48.1, 54.5) | 0.19 | 52.9 (48.3, 57.3) | 0.05 | | 49.7 (45.1, 54.2) | | 0.55 |
| **Parental Occupation** | | | | | | | | | | |
|  | Higher managerial, administrative and professional occupations | 41.3 (39.1, 43.6) | 48.1 (44.9, 51.3) |  | 36.7 (32.5, 41.0) |  | | 32.2 (28.2, 36.5) | |  |
|  | Intermediate occupations | 22.2 (20.4, 24.2) | 20.7 (18.3, 23.4) | <0.01 | 23.1 (19.6, 27.1) | 0.19 | | 24.3 (20.5, 28.6) | | 0.06 |
|  | Routine and manual occupations | 33.1 (31.0, 35.2) | 28.5 (25.7, 31.4) | <0.01 | 36.3 (32.0, 40.9) | 0.08 | | 39.1 (34.7, 43.6) | | 0.09 |
|  | Never worked | 3.4 (2.6, 4.4) | 2.7 (1.7, 4.2) | 0.11 | 3.9 (2.4, 6.3) | 0.55 | | 4.4 (2.9, 6.7) | | 0.21 |
| **Housing Tenure** | | | | | | | | | | |
|  | Own outright | 12.6 (11.3, 14.2) | 14.5 (12.4, 16.9) |  | 11.3 (8.9, 14.3) |  | | 10.1 (7.8, 13.1) | |  |
|  | Own with mortgage | 53.7 (51.4, 55.9) | 56.2 (53.0, 59.3) | 0.23 | 53.7 (49.2, 58.2) | 0.62 | | 48.5 (44.0, 53.1) | | 0.35 |
|  | Rent privately | 12.2 (10.8, 13.8) | 12.0 (10.0, 14.2) | 0.28 | 11.8 (9.1, 15.0) | 0.93 | | 13.1 (10.3, 16.6) | | 0.17 |
|  | Rent social housing | 21.5 (19.6, 23.5) | 17.3 (14.9, 20.0) | 0.02 | 23.2 (19.4, 27.6) | 0.65 | | 28.2 (24.2, 32.7) | | 0.04 |
| **Ethnicity** | | | | | | | | | | |
|  | White | 82.1 (80.1, 83.9) | 81.7 (78.8, 84.2) |  | 83.0 (78.9, 86.4) |  | | 81.9 (77.8, 85.3) | |  |
|  | Asian or Asian British | 9.1 (7.8, 10.7) | 9.3 (7.4, 11.6) | 0.24 | 9.6 (7.0, 13.0) | 0.96 | | 8.4 (6.0, 11.8) | | 0.16 |
|  | Black or Black British | 3.9 (3.1, 5.0) | 4.8 (3.5, 6.5) | 0.42 | 2.9 (1.5, 5.5) | 0.23 | | 3.3 (2.0, 5.4) | | 0.75 |
|  | Mixed ethnic group | 2.9 (2.2, 3.8) | 2.3 (1.5, 3.4) | 0.03 | 2.8 (1.7, 4.8) | 0.85 | | 4.1 (2.6, 6.6) | | 0.01 |
|  | Any other group | 2.0 (1.4, 2.8) | 2.0 (1.2, 3.3) | 0.48 | 1.7 (0.8, 3.7) | 0.59 | | 2.3 (1.2, 4.4) | | 0.18 |
| **Region** | | | | | | | | | | |
|  | England: North | 23.6 (21.7, 25.6) | 22.7 (20.2, 25.5) |  | 23.0 (19.4, 27.1) |  | | 25.9 (22.0, 30.2) | |  |
|  | England: Central/Midlands | 17.3 (15.7, 19.1) | 16.1 (14.0, 18.6) | 0.41 | 20.1 (16.7, 23.9) | 0.15 | | 17.0 (13.8, 20.7) | | 0.55 |
|  | England: South (incl. London) | 43.3 (41.0, 45.6) | 47.4 (44.2, 50.6) | 0.25 | 39.0 (34.6, 43.6) | 0.65 | | 39.2 (34.8, 43.9) | | 0.39 |
|  | Northern Ireland | 3.2 (2.9, 3.6) | 2.7 (2.3, 3.2) | 0.17 | 4.0 (3.3, 4.8) | 0.56 | | 3.5 (2.8, 4.3) | | 0.34 |
|  | Scotland | 7.8 (6.6, 9.1) | 6.9 (5.4, 8.9) | 0.08 | 8.4 (6.0, 11.5) | 0.18 | | 8.8 (6.6, 11.8) | | 0.58 |
|  | Wales | 4.8 (4.2, 5.5) | 4.1 (3.4, 4.9) | 0.04 | 5.6 (4.4, 7.1) | 0.06 | | 5.6 (4.3, 7.2) | | 0.75 |

*Weighted percentage of sample (%N) and 95% confidence interval (95% CI) are reported.

^†^ Multivariable logistic regression was used to assess the association between the level of daily UPF consumption and each sociodemographic characteristic.

The model was mutually adjusted for all sociodemographic characteristics, as well as total daily energy intake and relative energy from UPFs. Age was evaluated as a continuous variable and the remaining categorical variables were each compared to a reference level (sex - Female; ethnicity - White; parental occupation - higher managerial, administrative and professional occupations (higher occupations); housing tenure - own outright; region - England: North
